# RNA interference in marine and freshwater sponges: actin knockdown in *Tethya wilhelma *and *Ephydatia muelleri *by ingested dsRNA expressing bacteria

**DOI:** 10.1186/1472-6750-11-67

**Published:** 2011-06-16

**Authors:** Ajna S Rivera, Jörg U Hammel, Karri M Haen, Elizabeth S Danka, Brandon Cieniewicz, Ian P Winters, Dora Posfai, Gert Wörheide, Dennis V Lavrov, Scott W Knight, Malcolm S Hill, April L Hill, Michael Nickel

**Affiliations:** 1 Department of Biology, University of Richmond, Richmond, VA, USA; 2 Institut für Spezielle Zoologie und Evolutionsbiologie mit Phyletischem Museum, Friedrich-Schiller-Universität, Jena, Germany; 3 Iowa State University, Ames, IA, USA; 4 Department of Earth and Environmental Sciences & GeoBio-CenterLMU, Ludwig-Maximilians-University Munich, Germany; 5 Biology Department, College of the Pacific, Stockton, CA, USA; 6 Molecular Cell Biology, Washington University, St. Louis, MO, USA; 7 Molecular and Cellular Biology, Stony Brook University, Stony Brook, NY, USA; 8 Sigma-Aldrich Biotechnology, St. Louis, MO, USA

## Abstract

**Background:**

The marine sponge *Tethya wilhelma *and the freshwater sponge *Ephydatia muelleri *are emerging model organisms to study evolution, gene regulation, development, and physiology in non-bilaterian animal systems. Thus far, functional methods (i.e., loss or gain of function) for these organisms have not been available.

**Results:**

We show that soaking developing freshwater sponges in double-stranded RNA and/or feeding marine and freshwater sponges bacteria expressing double-stranded RNA can lead to RNA interference and reduction of targeted transcript levels. These methods, first utilized in *C. elegans*, have been adapted for the development and feeding style of easily cultured marine and freshwater poriferans. We demonstrate phenotypic changes result from 'knocking down' expression of the actin gene.

**Conclusion:**

This technique provides an easy, efficient loss-of-function manipulation for developmental and gene regulatory studies in these important non-bilaterian animals.

## Background

**Figure 1:**
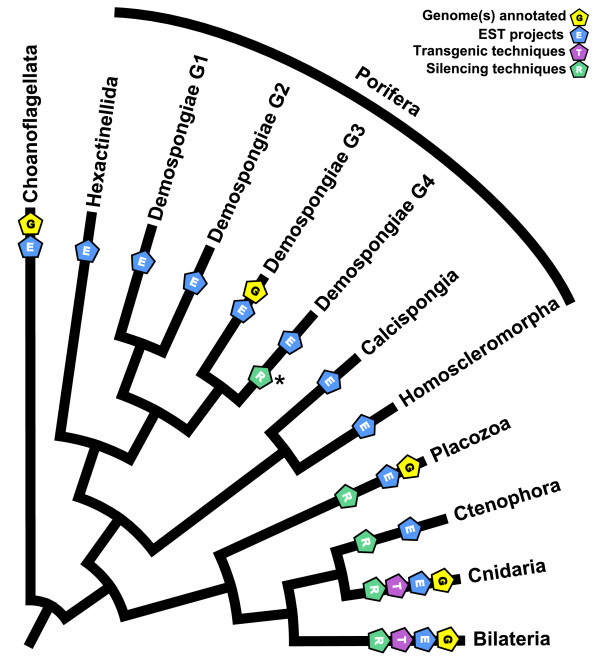
**Genomic resources and reverse genetics methods presently available for representative model species of non-bilaterian Metazoa**. Available methods for each group plotted on a cladogram (tree after [2,17]). While EST and/or genomic data are available for many representatives of each clade, functional (transgenic and silencing) techniques have only been adapted to a few non-bilaterian groups. The present study provides the first RNAi silencing protocols for a part of the Porifera, i.e. the two cultivatable Demospongiae G4 species *Ephydatia muelleri *and *Tethya wilhelma *(asterisk). There are no species currently maintainable in laboratory cultures among the demosponge clades G1 to G3, which would be an important prerequisite for future extensive RNAi studies.

The ability to reconstruct the evolution of animals relies heavily on the availability of comparative data to facilitate understanding of evolutionary trends from the molecular to the organismal level. Among the non-bilaterian animals, recent utilization of phylogenomics has increased our understanding of metazoan relationships [1-5]. Comparative genomic approaches were used to reconstruct ancestral genomes like the putative genome of the last common ancestor of the Metazoa (LCAM) [6-8]. Moreover, the same genomic data is used to evaluate hypotheses regarding the evolution of metazoan-specific pathways like those involved in development [9-11] or cell signaling [12-15]. For example, the genome of the demosponge *Amphimedon queenslandica *is playing a key role in deciphering evolutionary trends in Metazoan specific gene networks and pathways [e.g. [8,16]]. Nevertheless, evolutionary biology relies on more than genomic data. Functional studies based on reverse genetics techniques are needed to test the hypotheses provided by genomic analyses. However, the availability of genomic resources and reverse genetic techniques in the non-bilaterian animals is limited compared to the other animals (Figure 1, tree after [2,17]). For the Cnidaria, the Placozoa, and the Ctenophora genomic data and reverse genetic techniques are available [e.g. [18-22]]. However, thus far, only some genomic data is available for the Porifera, and the sister group of Metazoa, the Choanoflagellata. Reverse genetic techniques for these animal groups is of high priority for a comprehensive analysis of evolution and development of the non-bilaterian Metazoa.

Apart from permanent knockout and/or transgenic lines [e.g. [23]], RNA interference (RNAi) is a powerful tool to alter the expression of genes [24-26]. RNAi can be mediated in a variety of ways, for example soaking animals in double-stranded RNA (dsRNA) or morpholinos [18,19,27], injecting dsRNA or morpholinos [28,29], or feeding dsRNA expressing bacteria [24,25,30]. Among these techniques, certain limitations have to be considered: (1) Soaking animals in dsRNA appears to be most problematic in seawater due to issues of RNA stability. This might be one of the reasons why functional genomic studies in marine non-Bilateria are scarce. So far, soaking techniques have only been adapted to *Eleutheria dichotoma, Aiptasia pallida *and *Trichoplax adhaerens *[18,19,27]. In some studies, the salinity has been altered [18,19], which cannot be tolerated by most marine organisms. (2) The injection of dsRNA or morpholinos is only efficient if early embryos can be investigated. (3) Feeding of dsRNA-expressing bacteria requires efficient uptake of bacteria either naturally or by mimicking other food sources [30].

Because sponges represent a key group for our understanding of Metazoan evolution including the changes in gene regulation that took place leading to multicellularity and complex body plans in this lineage, our aim was to test and establish RNAi protocols in one freshwater and one marine sponge species. By addressing both living modes we intended to provide a broad basis for future adaptations of this method to other sponge species.

The marine species *Tethya wilhelma *Sarà, Sarà, Nickel & Brümmer, 2001 (Demospongiae, Hadromerida, Tethyidae) and the freshwater species *Ephydatia muelleri *(Lieberkühn, 1856) (Demospongiae, Haplosclerida, Spongillidae) are emerging basal animal model organisms. *Ephydatia *is an abundant and common sponge found on all major land masses in the world [31]. Recent research has lead to a better understanding of *Ephydatia *biology including cellular physiology and function as well as the role of conserved signaling pathways [e.g., [32-35]]. One of the most important aspects is that during winter *Ephydatia *forms thousands of gemmules which are cysts containing totipotent stem cells (archeocytes). These gemmules can be collected, stored at 4°C for up to one year, and easily induced to hatch into fully differentiated adults on the laboratory bench. This cultivation routine provides a system to study stem cell differentiation and animal body plan development in these animals [32,36]. A genome and a transcriptome are currently in the process of being sequenced for this species. *Tethya wilhelma *is one of the few marine sponges that can currently be permanently maintained in the laboratory based on its capability to reproduce asexually through buds [37-39]. In addition, this species has been studied on a broad scale including functional anatomy [40-42], contractile and movement behavior [13,37,43], physiology of integration [13,44,45] and bud development [39]. A transcriptome has been sequenced recently and is currently being annotated, and sequencing of the genome is in the planning stage.

To enable functional studies in sponges we developed a protocol for bacterial mediated RNAi-induced silencing based on gene silencing techniques developed for *Caenorhabditis elegans *[24,25]. The protocol has been adapted to the marine and freshwater environments and tested by knocking down cytoplasmic actin mRNA levels in juvenile specimens of *T. wilhelma *and *E. muelleri*. Our results indicate that this pathway is able to function in both marine and freshwater sponge species.

## Results and Discussion

### Actin sequences used for knockdown experiments

From a *Tethya *cDNA library, we identified a 707 bp fragment of *T. wilhelma *as a putative beta-actin ortholog (*Tw-actb*; Additional File 1, Figure S1). The *Tw-actb *fragment contains a putative 5'UTR (nt 1-126) and the 5' part of the putative coding sequence (CDS) (nt 127-705). This sequence is quite similar to the *Homo sapiens *β-actin sequence (NM_001101 [46]) with 73.4% pairwise identities overall. A motif search in the translated putative CDS region of *Tw-actb *revealed PROSITE [47] Actin signature 1 (PS0046) and 3 (PS01132) at residues 54-64 and 105-117 respectively. Actin signature 2 (PS00432) has not been identified; it may reside in the uncloned 3' terminus. Using beta-actin degenerate PCR primers, we isolated a 338 bp fragment of *E. muelleri *as a putative beta-actin ortholog (*Em-actb*; Additional File 1, Figure S1). The *Em-actb *fragment is 100% identical to the putative *Tethya *beta-actin ortholog at the amino acid level. Our *Em-actb *fragment also contains PROSITE actin signature 3. Both fragments were cloned into the L4440 plasmid (Additional File 1, Figure S2) and transformed into *Escherichia coli *HT115(DE3), which was induced to produce dsRNA.

### *In silico *evaluation of the actin sequences used for knockdown experiments

In order to ensure that we are targeting cytoplasmic actin and not another actin isoform or actin-related protein, we performed an *in silico *analysis. Using species specific BLAST searches [48] we translated both *E. muelleri and T. wilhelma *actin coding sequences and aligned them to other actins. These actin sequences were obtained from a protein sequence alignment used in a previous study [49] as well as contig translations (see Additional File 1, Figure S1, Additional File 1, Figure S2) based on tblastn searches using *H. sapiens *β-actin (AAH16045) against the following transcriptomes housed at NCBI dbEST [50]: *Monosiga ovata *(Choanoflagellata), *Amphimedon queenslandica *(Porifera, Demospongiae), *Leucetta chagosensis *(Porifera, Calcarea), *Oscarella carmella *(Porifera, Homoscleromorpha), *Nematostella vectensis *(Cnidaria, Anthozoa), *Hydra vulgaris *(Cnidaria, Hydrozoa), *Mnemiopsis leidyi *(Ctenophora), and *Trichoplax adhaerens *(Placozoa). The RaxML tree we generated from these data showed that both *T. wilhelma *and *E. muelleri sequences *clearly group within all other metazoan putative cytoplasmic Actins and are clearly separated from other Actins or Actin-related proteins (Additional File 1, Figure S3). An alignment of the partial coding sequences of *Tw-actb *and *Em-actb *used for RNAi with 9 additional holozoan orthologs produced pairwise identity of 82.4% on the nucleotide level (Additional File 1, Figure S4) and 97.2% on the amino acid level (Additional File 1, Figure S5). RaxML tree and alignment analyses confirm that cytoplasmic actin was targeted in our RNAi knockdown experiments and thus we expect cytoplasmic actin specific phenotypic effects.

### Evaluation of a soaking method for dsRNA delivery

We first attempted a soaking method for dsRNA delivery in *E. muelleri*. As negative controls, we used dsRNA for green fluorescent protein (GFP) since sponges do not express GFP. We also used double-stranded DNA (dsDNA) generated by PCR amplifying the *E. muelleri *beta-actin gene since dsDNA should not be processed through the RNAi pathway.

Soaking sponges in high concentrations (> 50 μg/mL) of any nucleic acid (GFP dsRNA, actin dsRNA, actin dsDNA) leads to potentially cytotoxic effects to the sponges. Freshwater sponges are sensitive to changes in osmolarity [51], which could be the cause of the observed cytotoxicity. When exposed to high nucleic acid concentrations, the tissues regressed, choanocyte chambers seemed to collapse, and sponges ceased pumping. Thus, in titration experiments we tested for concentrations at which dsDNA and GFP dsRNA would not have an observable effect on sponge morphology, while actin dsRNA would. A concentration of 1-10 μg/mL of beta-actin dsRNA led to distinct observable morphologies; including loss of contact of the growing edge of tissue with the culture plate, loss of definition in the canal structure of the sponge, and poorly defined choanocyte chambers (Additional File 1, Figure S6A,B,C). Longer exposures to beta-actin dsRNA resulted in more pronounced phenotypes (Additional File 1, Figure S6D,E,F). Control experiments with exposure to GFP dsRNA or beta-actin dsDNA at similar concentrations, did not exhibit phenotype changes (data not shown). Interestingly, removal of the dsRNA beta-actin solution and replacement with normal culture media lead to full recovery of the sponge tissue after one to two days including canals, distinct connections to the culture plate and a developed osculum (Additional File 1, Figure S6G,H,I,J).

Our data indicate that a soaking method is viable for induction of the RNAi pathway in *E. muelleri*. However, the tight range in the amount of dsRNA that seemed to be effective to produce an expected phenotype will require titration experiments for each gene addressed in future RNAi studies. We did not attempt a soaking method for *T. wilhelma *since it is necessary to alter the salinity (by a decrease of ~50%) of the culturing media in order to ensure RNA stability. We know that *T. wilhelma *and most other marine sponges do not tolerate salinity changes in this range without experiencing changes in contractile behavior and most likely phenotypic changes (JH & MN, unpublished observations). Thus, a soaking method would likely not be viable for marine species.

### Feeding method for the freshwater sponge *Ephydatia muelleri*

**Figure 2:**
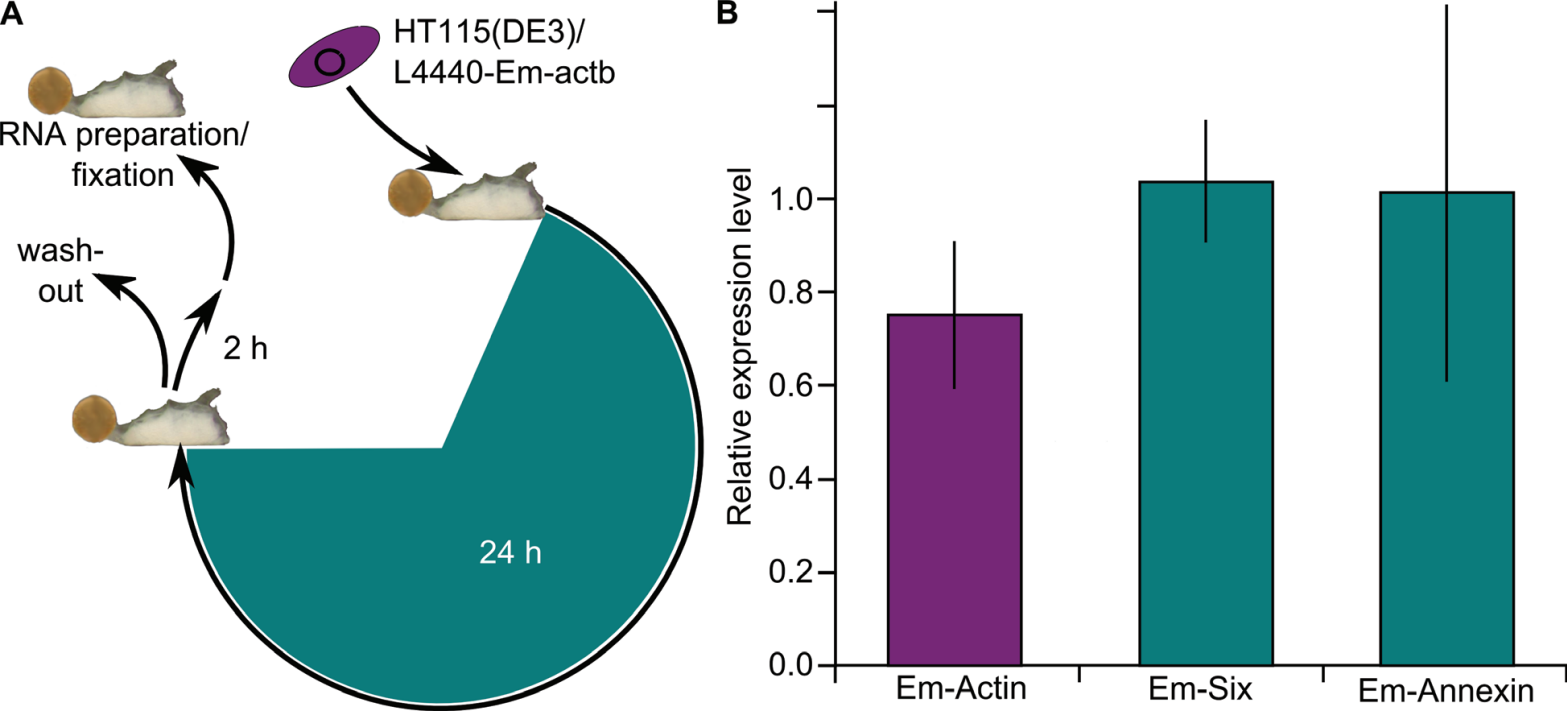
**Experimental feeding scheme and quantitation of actin expression levels after RNAi treatment in *Ephydatia muelleri***. **A**. After feeding for 24 hours with induced HT115(DE3)/L4440-Em-actb or non-induced controls, sponges were washed several times in 1X Strekal's medium, photographed, and harvested for RNA preparation or fixation within 1-2 hour. **B**. qRT-PCR assay for gene expression levels of Em-actin (purple bar) and two control genes. (Em-six and Em-annexin, green bars) after dsRNA treatment with induced HT115(DE3)/L4440-Em-actb. Levels were normalized to Ef1α and gene expression levels are given as relative levels of RNAi treated sponges against control sponges. Combined data with N_Em-actin _= 11, N_Em-Six _= 3, N_Em-Annexin _= 3; bars represent standard deviations.

Since sponges naturally feed on bacteria, a feeding method for dsRNA delivery might be more universally applicable in marine and freshwater sponge systems. In *E. muelleri*, we fed sponges HT115(DE3)/L4440 *E. coli *expressing *Em-actin *dsRNA, for 24 hours. Afterwards, sponges were washed several times in culture media to remove bacteria and tissue was harvested within one to two hours (Figure 2A). Sponges were also fed HT115(DE3) or bacteria expressing GFP to monitor feeding behavior and to test for a response to induced bacteria. GFP-fed sponges did not show any response that differed from sponges fed bacteria lacking expression vector (data not shown).

To test the ability of actin dsRNA to reduce actin expression, we performed quantitative-RT-PCR (qRT-PCR) assays. For qRT-PCR analysis, we fed sponges either dsRNA-expressing bacteria, untransformed bacteria, or GFP-expressing bacteria for 24 hours. Sponge tissue was harvested and we utilzed qRT-PCR to access actin expression levels. A relative quantification procedure was used to calculate a ratio of treatment to control expression levels, either for the gene of interest (i.e., *Em-actb*) or for the non-target genes (i.e., *Em-six *and *Em-annexin*). If the target gene was not affected by actin RNAi we would expect a ratio of 1. The ratio of paired treatment and control expression levels were averaged and are shown in Figure 2B. We performed eleven total qRT-PCR experiments with L4440-*Em-actb *(abbreviated *dsEm-actb*)-fed sponges and controls. In ten of these experiments, *Em*-*actb *levels were lower in dsRNA-fed sponges than controls, when normalized to the housekeeping gene *EF1-alpha *expression levels. These results were significant (p < 0.05) as the relative *Em-actb *mRNA expression levels dropped down to 75% of the control values (thus, average knockdown in RNAi-fed sponges was near 25%, with a maximum knockdown value of ~55%). Longer treatments (48 hours) with the same bacteria did not lead to a greater effect (data not shown).

To test for nonspecific effects, we repeated the qRT-PCR experiment using sponges fed bacteria expressing ds*Em-actb *and then assayed expression levels for *Em-annexin *and *Em-six1/2*. Em-Annexin is a structural protein expressed in the archaeocytes, and in developing and mature choanocyte chambers [36] while Em-Six is a transcription factor expressed in multiple cell types including archaeocytes and choanocytes throughout development (AH, unpublished data). Of three separate experiments totaling about 15 sponges per condition, *Em-annexin and Em-six *mRNA levels were not significantly different in *dsEm-actb*-treated sponges compared to HT115(DE3)-fed control sponges (Figure 2B). Any differences were small and non-directional, suggesting that ds*Em-actb *did not affect *Em-annexin *or *Em-six *expression.

To further demonstrate, on the molecular level, that it is possible to knockdown genes other than actin, we present RT-PCR, qRT-PCR and Western blot data for experiments conducted where sponges were either fed bacteria or soaked with dsRNA directed to *Em-annexin *and *Em-six *genes. We used RT-PCR and gel-based analysis to evaluate *dsEm-annexin*-treated sponges using both the soaking (sponges soaked in 10 μg/mL *Em-annexin *dsRNA) and feeding-based methods (described above). In both cases, reduced expression of *Em-annexin *is apparent when compared with control-treated sponges (Additional File 1, Figure S7A). We used qRT-PCR to demonstrate that sponges fed HT115(DE3)/L4440 *Em-six *dsRNA constructs showed reduced *Em-six *expression when compared to control-treated tissues. These results were significant and the relative *Em-Six *mRNA expression levels dropped down to ~69% of the control values (thus, average knockdown in RNAi-fed sponges was ~31%; with a maximum knockdown value of ~70%) (Additional File 1, Figure S7B). It is clear that several parameters will need to be optimized to consistently achieve > 50% knockdown. These include time and length of exposure to dsRNA, age of sponges and ability to consistently induce bacteria and control for amount of bacteria fed to sponges.

To demonstrate that knockdown is also evident at the protein level, we performed Western blot analysis for both Actin and Six proteins after treatment with either *dsEm-actb *or *dsEm-six *RNAi (Additional File 1, Figure S8). The variation in knockdown levels for Actin protein found in Western analysis was consistent with the variation in gene expression levels found by qRT-PCR after *dsEm-actb *treatment. Two different representative Western blots showing *dsEm-actb*-treated tissue compared to control-treated tissue are shown and illustrate that Actin protein levels are lower after *dsEm-actb *treatment (Additional File 1, Figure S8A). In response to *dsEm-six *treatment, protein levels for Six were also reduced while the same blot showed that Actin protein levels remain relatively constant (Additional File 1, Figure S8B).

### Feeding method for the marine sponge *Tethya wilhelma*

**Figure 3:**
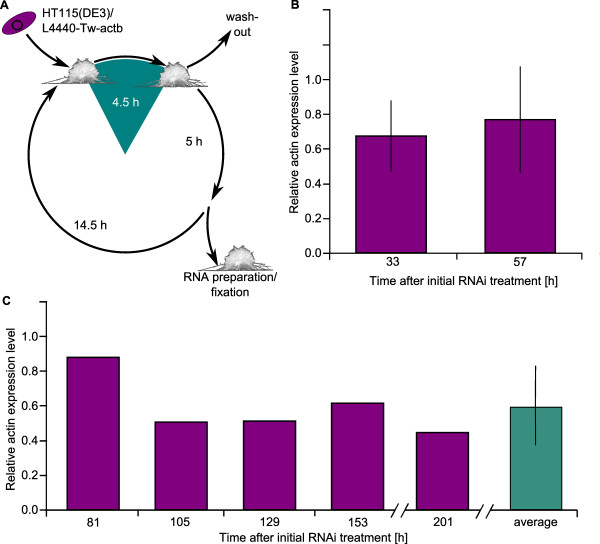
**Experimental feeding scheme and quantitation of actin expression levels after RNAi treatment in *Tethya wilhelma***. **A**. After pulse feeding with induced HT115(DE3)/L4440-Tw-actb or non-induced controls for 4.5 hours and subsequent wash out by fresh ASW, sponges were cultivated in ASW for 19.5 hours. Every day, after the pulse feed, some of the sponges were separated out, cultivated for 5 hours, and washed in seawater until they were harvested for RNA preparation and fixation. **B - C**. qRT-PCR assay for gene expression levels of *Tw-actb *(purple) after dsRNA treatment with induced HT115(DE3)/L4440-Tw-actb. Levels were normalized to RPS5 and gene expression levels are given as relative levels of RNAi-treated sponges against control sponges [69]. **B**. Short-term actin knockdown: relative actin-mRNA levels in *T. wilhelma *specimens sampled at 33 hours (N = 3) and 57 hours (N = 3) after initial pulse-feeding. Bars represent standard deviation (N = 3). **C**. Independent long-term actin knockdown experiment: actin expression in single *T. wilhelma *specimens after daily 4.5 hour pulse-feeding with dsRNA-expressing HT115(DE3)/L4440-Tw-ctb *E. Coli *over 8 days. Levels of single specimens were normalized as above (purple; each bar represents a single specimen); average relative actin expression (green; with N = 5).

Using the same bacteria and a similar expression vector construct as described above, but a different feeding profile (Figure 3A) we fed *T. wilhelma *specimens *Tw-actb *dsRNA expressing HT115(DE3). Larger specimens of *T. wilhelma *were cultured over a period of 33 and 57 hours, respectively, and pulse-fed with ds*Tw-actb *expressing bacteria or a control strain for 4.5 hours each day. This resulted in actin knockdown in RNAi-treated sponges (Figure 3B). After 33 hours of cultivation, including two pulse-feeding cycles, relative *Tw-actb *expression levels dropped down to 67.5% in relation to control animals, representing an average knockdown by 32.5%. After an additional dsRNA feeding pulse within a total cultivation time of 57 hours, the relative actin mRNA levels remained within a reduced range of 76.9% in relation to control animals. Thus, all ds*Tw-actb*-treated specimens (N = 6) showed significant reductions compared to endogenous mRNA levels (dependent t-test, p < 0.05). In contrast, pulse feeding of ds*Tw-actb *expressing bacteria for a reduced time of 20 minutes did not reveal any knockdown effect within 33 or 57 hours (data not shown) for this species.

To determine if greater knockdown is observed in *T. wilhelma *after longer-term exposure to *dsTw-actb*, we repeated the experiment over a period of eight days. Between 81 and 201 hours after initial feeding, single specimens were tested for relative *Tw-actb *expression levels, which were between 87.6% (at 81 h) to 50.4% (at 105 h) to the level of *Tw-actb in *control sponges. While this is a wide range, the average (68.7% ± 23%; N = 5), is close to the total average of the short-term experiment (72.2% ± 24%; N = 6), which represents significant reductions in expression levels (dependent t-test, p < 0.05).

To determine viability of sponges affected by long-term actin knockdown, all specimens that were used for quantification in the long-term experiment were time-lapse imaged during the 24-hour period preceding sampling and preparation for RNA quantification. All specimens displayed typical contractile behavior (Additional File 1, Figure S9, Additional File 2) demonstrating the general viability of the RNAi-treated specimens. Thus, our results demonstrate that daily pulse feeding for 4.5 hours does not result in lethal effects as it has been observed by direct dsRNA incubation in a dose-dependent manner [27].

#### Evaluation of morphological changes in response to actin 'knockdown' in Ephydatia muelleri

**Figure 4:**
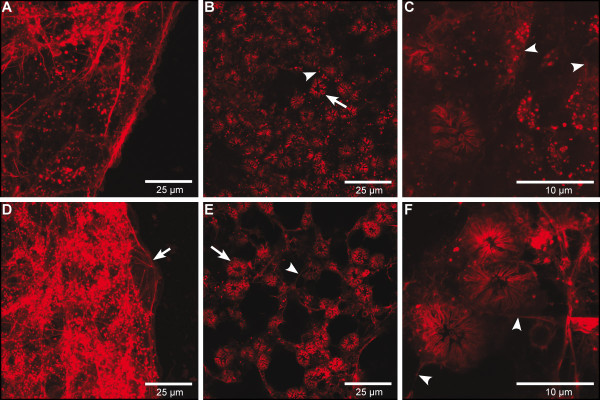
**Phalloidin-stained tissue in control vs. actin RNAi-treated *Ephydatia muelleri***. Confocal laser scanning microscopy images of BODIPY 558/568 phalloidin conjugate-stained whole-mount preparations of *E. muelleri*. **A - C**. Actin RNAi-treated sponge tissues. **D - F**. Control-treated sponges. A and D. Arrow shows Actin filament connections to substrate. These connections are generally missing in RNAi-treated tissues. B and E. Arrows show choanocyte chambers while arrowheads point to Actin connections (in endopinacocytes) between chambers, which are largely missing in treated sponges. C and F. Arrowheads show extensive Actin connections between and around choanocyte chambers. These connections are missing or not as extensive in treated tissues.

To examine morphological changes due to actin knockdown in *E. muelleri*, we stained ds*Em-actb*-treated sponges and control sponges with Bodipy-558/568 conjugated phalloidin. We examined three major regions of the sponge tissue by confocal laser scanning microscopy (cLSM): 1) the outer edge of sponge growth, 2) the regions containing newly developed choanocyte chambers, and 3) the endopinacocyte-lined canals surrounded by choanocyte chambers. An extensive Actin network was present at the edge of sponge growth. The outer surface of the sponge that forms attachments to the substrate is composed of exopinacoderm and basopinacoderm and is made of continuous epithelium where some connective and migratory (amoebocytes) cells are observed. The region also contains filopodia of multipolar cells leading from the mesohyl to the exopinacoderm. Small foci represent the edge of two abutting cells when viewed from above, and larger foci likely represent focal adhesion points where Actin bundles highlight filopodia of mesohyl cells. Sponges fed ds*Em-actb *have a less extensive Actin network at the growing edge of the sponge. Many fibers do not run continuously through the sponge tissue and the continuous fibers often lack the network organization seen in control sponges. These data likely indicate that there is a reduced cellular mobility in the mesohyl (Figure 4 A&D).

The sponge region containing newly developed choanocyte chambers (choanosome) was chosen as it is a relatively thin region but allows us to see the overall pattern of developed sponge tissue. In this region the Actin-rich choanocytes themselves look very similar in treated and control sponges. To further investigate, we performed transmission electron microscopy on a control and a *dsEm-actb*-treated sponge to see whether ultrastructural aspects of choanocytes were affected after RNAi treatment. While a more thorough sampling strategy is required, we found one trait in the microvillar collar that may differ between treatment and control sponges. Actin RNAi-treated choanocytes had a greater percentage of cells with misshapen/folded collars (Additional File 1, Figure S10). The control sponges, on the other hand, contained numerous perfectly round collars, which were not observed in the Actin RNAi-treated sponges (Additional File 1, Figure S10). However, the pattern of endopinacocytes connecting the choanocytes differed markedly. Control sponges have canals that are clearly lined by Actin-rich endopinacocytes. ds*Em-actb*-fed sponge canals are far less clearly lined (Figure 4 B&E). In ds*Em-actb*-treated sponges, the endopinacocytes that span the spaces between choanocytes are thin and display a weaker Actin signal.

The Actin cytoskeletal network is thus reduced in ds*Em-actb*-fed pinacocytes, but the choanocytes appear to be unaffected at the level observed with light microscopy. In the canals lined by Actin-containing pinacocytes, the Actin fibers run continuously around the canals, over the span of many cells. Control sponges have clearly outlined canals in most focal planes. ds*Em-actb*-fed sponges, however, have canals that are not as well-defined and are lacking clear phalloidin-stained pinacocytes in many planes (Figure 4 C&F). Overall, our observations suggest a general knockdown of the Actin cytoskeleton with a less prominent Actin network.

#### Evaluation of morphological changes in response to actin 'knockdown' in Tethya wilhelma

To examine morphological changes due to actin knockdown in *T. wilhelma*, we fixed sponges that had been pulse-fed induced HT115(DE3)/*Tw-actb *bacteria expressing dsRNA and control HT115(DE3) bacteria daily (Figure 3) after 33 and 57 hours. Using whole-mount preparations we examined two major regions of the sponge by cLSM that were stained using Alexa-488-conjugated phalloidin: 1) the exopinacocyte, and 2) the cortical mesohyl.

**Figure 5:**
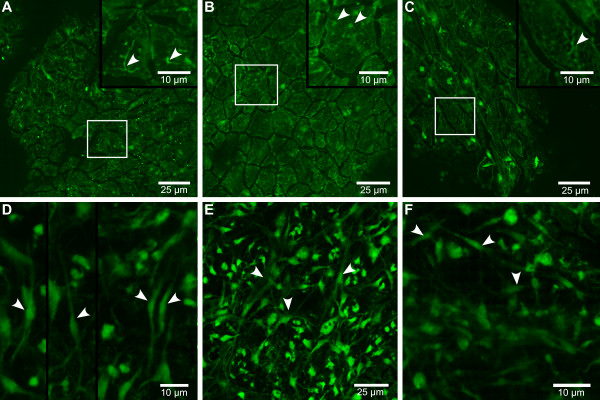
**Phalloidin-stained tissue in control vs. actin RNAi-treated *Tethya wilhelma***. Confocal laser scanning microscopy images of Alexa 488 phalloidin conjugate-stained whole mount preparations of *T. wilhelma*. **A, D and F**. Control treated sponges. **B and E**. Actin RNAi treated sponges 33 hours after initial feeding (see feeding scheme in Figure 3). **C**. Actin RNAi treated sponge tissues 57 hours after initial feeding. A - C. Exopinacoderm layers showing pinacocytes. Insets display details of the main image. Arrowheads show Actin ripples at the cell surface as parts of the pinacocyte Actin network. Note: due to slight hypoosmotic fixative in order to avoid cell shrinking, the sponges are slightly inflated during fixation, forming small artifactual gaps between pinacocytes due to their extremely thin contact zones between the pinacocytes. This slight artifact is systemic in all samples and does not interfere with the characters important in the context (see Additional File 1, Figure S11 for more details). D. Three detail images of bipolar (moving) mesohyle cells (arrowheads) in control sponges. E - F. Multipolar cells (arrowheads): hypertrophic, large cells in RNAi-treated sponges (E) and normal multipolar cells in control sponges (F).

Phalloidin staining revealed an extensive Actin network in the exopinacocytes with Actin-rich ripple-like structures on the surface of the exopinacocytes (Figure 5A), structures typical in *T. wilhelma *and resembling lamellipodia extensions of fibroblasts (see Additional File 1, Figure S11). In the ds*Tw-actb*-treated specimens, after 33 hours the exopinacocytes showed fewer Actin-rich ripples (Figure 5B) than in the control, while after 57 hours, ripples were almost absent (Figure 5C). In addition, the Actin network was overall less prominent in the treated sponges than in the control specimens.

The largest effect was observed in the cortical mesohyl. The control sponges showed a large number of extremely long spindle-shaped bipolar cells (Figure 5D), which are typical morphotypes of highly mobile cells [52] 'frozen' in movement by fixation. In contrast, the ds*Tw-actb*-treated sponges were characterized by atypical, extremely large atrophic multipolar cells (Figure 5E) and only few bipolar cells. In comparison, multipolar cells in the control sponges were much smaller (Figure 5F).

The changes in the pinacocytes with the reduced Actin-rich lammellae and atypical mesohyle with the atrophic multipolar cells and the lack of bipolar cells can be explained by decreased actin expression. While the Actin turnover rate is not high enough to produce lethal effects or cessastion of contractility, processes based on Actin dynamics like cell mobility or lamellipodia formation seem to be strongly affected. Since the atrophic multipolar cells correlate with a lack of mobile bipolar cells, it seemed that both forms actually represent extreme morphotypes of a single archeocyte-like cell type.

## Conclusions

For the first time, we report successful RNAi techniques for gene knockdown in the sponges *E. muelleri *(freshwater) and *T. wilhelma *(marine). We used dsRNA soaking methodology for *E. muelleri *and a bacterial feeding method adapted to freshwater and marine culture media for both species. Our experiments demonstrate a significant reduction of endogenous mRNA levels in both species after feeding and represent the successful proof-of-principle for bacteria-mediated feeding induced RNA interference in marine and freshwater demosponges. We tested dsRNA methodology using cytoplasmic actin orthologs, a ubiquitously expressed gene involved in cell shape and motility.

In *E. muelleri*, qRT-PCR analysis showed a modest (~25%) but significant (p <0.05) knockdown of *Em-actb *after feeding sponges bacteria expressing dsRNA against *Em-actb *for 24 hours compared to controls, with a maximum knockdown of about 55%. *T. wilhelma *sponges treated for 33 or 57 hours showed similarly modest but significant (p < 0.05) reductions. *T. wilhema *treated for up to 8 days showed actin mRNA reductions throughout the entire period of the experiment. The maximum knockdown caused by ds*Tw-actb*-expressing bacteria was almost 50% compared to the control-group mRNA level. We also demonstrated in *E. muelleri*, at both the mRNA and protein level, that it is possible to knock down additional genes (*Em-annexin *and *Em-six*), thus illustrating this method should be applicable for other target genes.

The reduction in expression for actin demonstrates an efficient silencing for a highly expressed gene that may account for about 5% of the total protein content of a cell [53]. Furthermore, in order to increase the level of reduction, longer periods of treatment with dsRNA might be required for highly expressed genes or stable proteins like Actin. Hints of this were seen in the long-term *T. wilhelma *feeding and *E. muelleri *soaking experiments (Figure 3C, Figure S6, Additional File 1). It is possible that the RNAi effect may be greater than measured, because the knockdown effect may only be measured in surviving cells. Sponges are known to have high proliferation activity, a short cell cycle duration and cell shedding that is predominantly confined to the choanocytes [e.g. [54]]. This high cell turnover rate may be part of the reason that actin (or probably any gene) will not be knocked down at great levels. Due to the biology of these organisms and the method used, feeding sponges dsRNA will likely be a technique that can be utilized to knockdown, but not knockout gene expression. However, the overall significantly reduced mRNA levels during the entire period of the experiment clearly demonstrated the potential of bacterial-mediated RNAi-induced silencing for functional gene knock down studies in poriferans.

After feeding *Em-actin *dsRNA, *E. muelleri *did not display a macroscopic phenotype that could be clearly described at the cellular level. However, the primary Actin knockdown phenotype shown by phalloidin staining was a less extensive Actin network that was manifested in a "crumpled" edge, where tissues should be adhering to the substrate and migrating outwards. Additionally, a reduction of cellular connections in the choanocyte-rich regions and canals due to loss of Actin in endopinacocytes was observed. *T. wilhelma *showed neither a reduced survival rate nor alterations of rhythm or kinetics of body contractions. Like in *E. muelleri*, RNAi treated specimens of *T. wilhelma *displayed no distinct macroscopic phenotype. However, we found strong effects on mesohyle and pinacoderm cell anatomy of *T. wilhelma*. Apart from reduced Actin content and altered Actin network appearance in pinacocytes, the most prominent effects were hypertrophic mesohyle cells and the lack of mobile bipolar cells, indicating a reduced mobility and cell shape dynamics in dsRNA-treated specimens.

RNAi methods allow us to begin to study sponge gene function in a directed manner, including questions regarding evolution of gene regulatory networks, nervous system and muscle evolution, and also functional studies concerning biomineralization and other metabolic processes. Our results also suggest that RNAi might be a valuable tool to investigate cell-type functionality in sponges, as has been shown for other organisms, like *Hydra *[e.g. [30]]. This would facilitate studies on the evolution of cell differentiation in sponges. Additionally, it might help us to differentiate variable morphotypes of one single cell type versus other cell types. Thus, RNAi would help solve a long-standing problem for sponge biologists and evolutionary biologists interested in cell type evolution [e.g. [55]] by allowing unique studies to clearly define and distinguish sponge cell types, most likely in conjunction with in situ hybridization. We assume that our protocol will be readily applicable to other marine sponge species like the homoscleromorph *Oscarella lobularis *[56,57] or the calcareous *Sycon ciliatum *and *S. raphanus *[58-60]. It will also be adaptable to the freshwater species *E. fluviatilis *and *Spongilla lacustris*, which are also commonly used to study sponge biology [36,61-63]. In conclusion, our present study closed an existing gap for a functional genomics protocol for the Porifera and will thus stimulate a number of comparative functional studies, which will deepen our understanding of metazoan evolution.

## Methods

### Sponges

*E. muelleri *gemmules were harvested from sponges collected from a dam outflow near Griswold, Connecticut, USA (41°35'4"N, 71°55'15"W). Gemmules were washed in 3% hydrogen peroxide, and re-washed several times in sterile, cold, 1X Strekal's [64] medium and stored at 4°C in the dark until use. Washed gemmules from the freshwater sponge *E. muelleri *are placed in 12-well plates containing 2 mL of 1x Strekal's medium and kept in the dark at ~25°C for hatching. After hatching (2-3 days after plating) the 1x Strekal's was replaced daily. Gemmules were allowed to develop to stage 5 before they were treated with either dsRNA or fed bacteria to ensure that feeding structures had fully developed.

*T. wilhelma *originated from its type locality, the aquarium of the Zoologisch-Botanischer Garten Wilhelma Stuttgart (Germany) [38]. Due to its asexual reproduction by budding *T. wilhelma *was permanently cultured in the laboratory in Jena in tropical seawater aquaria under constant moderate flow conditions at a temperature of 24°C and a light regime of 12 h day and night. Artificial seawater (ASW; Tropic Marine, Wartenberg, Germany; dissolved in demineralized water) was partly renewed every few weeks. During cultivation, sponges were fed on a daily basis using finely dispersed commercial aquarium invertebrate food ('Artificial Plankton' Aquakultur Genzel, Freiberg am Neckar, Germany). For experiments, we used fully grown sponges ('adults') and stage-4 buds around 1 mm diameter, which were released shortly before the experiment, as well as juvenile sponges of up to 3 mm [for details on bud developments see ref. [39]].

### EST library construction and sequencing

Several specimens of *T. wilhelma *were sampled from the aquarium of the Zoologisch-Botanischer Garten Wilhelma Stuttgart (Germany) and immediately frozen in liquid nitrogen. They were stored at -80°C and shipped on dry ice to Iowa State University (Ames, Iowa, USA). Immediately after arrival, the samples were stored at -80°C until they could be re-immersed in liquid nitrogen and ground with an RNase-free mortar and pestle. The following protocols for RNA isolation and cDNA synthesis used reagents from Invitrogen (Carlsbad, CA, USA): 1 mL of TRIzol Reagent was added for every 50 mg of ground tissue, and total RNA extraction was carried out according to the manufacturer's instructions. Resultant RNA pellets were dissolved in 25 μL of DEPC-treated water, with the addition of 1 μL RNasin RNA protectant, and checked for RNA integrity on an agarose gel. mRNA was isolated from total RNA aliquots using the FastTrack MAG mRNA Isolation Kit with a Magna-Sep magnetic particle separator. mRNAs were treated with Calf Intestinal Phosphatase (CIP) to dephosphorylate uncapped 5' ends and processed with the GeneRacer kit for first-strand synthesis of full-length cDNAs using SuperScript III reverse transcriptase (RT). Second-strand synthesis was carried out using standard kit primers and Platinum Taq HiFi DNA polymerase. Size fractionation of cDNAs was achieved by gel-extracting cDNA pools of successive sizes with the PureLink kit. cDNA pools were then treated for blunt-end ligation and cloned with the Zero Blunt TOPO PCR Cloning Kit for sequencing. Random clone libraries were grown overnight in 96-well culture plates and submitted to the Iowa State University DNA Sequencing Facility for automated plasmid extraction and sequencing of cDNA inserts. Resulting sequence data was queried against the NCBI GenBank for identification of cDNA sequences with known function.

#### Maximum likelihood gene tree analysis

Using the cloned *Tw-actb *and *Em-actb *sequences, blastn searches were performed in a recently constructed EST database (*T. wilhelma*) and in Genbank (*E. muelleri*, organism-specific search). The dataset was complemented by organism-specific tblastn searches using *H. sapiens *β-actin (AAH16045). Contigs were created using Geneious Pro 4.74 (Biomatters, Auckland, NZ) using the assembly function; assemblies were visually inspected and corrected manually for errors caused by the automatic assembly. The contig sequence was analyzed for the actin open reading frame (ORF) starting with the amino acid sequence MDDXVAALV. The translated ORF was added to a previously published actin and actin-related protein amino acid sequence alignment [49]. Sequences were submitted to Genbank (*T. wilhelma*: HQ693078, *E. muelleri *HQ677599).

Additional cytoplasmic actin contigs and amino acid translations (see Additional File 3, Additional File 4) were created in the same way by organism-specific tblastn searches using *H. sapiens *β-actin (AAH16045) for the following early-branching Metazoa or Holozoa, included in the alignment: *Amphimedon queenslandica*; *Hydra vulgaris*; *Leucetta chagosensis*; *Mnemiopsis leidyi*; *Monosiga ovata*; *Nematostella vectensis*; *Oscarella carmela*; *Oopsacas minuta*; *Trichoplax adhaerens*.

The existing amino acid alignment was expanded using the Muscle algorithm of Geneious Pro. A rapid bootstrap analysis [65] on 100 replicates followed by search for the maximum likelihood tree was performed using RAxML 7.2.6 (PTHREADS) with the GTR (General Time Reversible) amino acid substitution model with site-specific evolutionary rates (4 discrete GAMMA categories). RAxML was called as follows: ./raxmlHPC-PTHREADS-SSE3 -T 4 -f a -x 12345 -p 12345 -# 100 -m PROTGAMMAGTR -o At-arp4. The unrooted gene tree was visualized using Geneious Pro.

#### RNAi constructs

PCR primers for producing dsRNA for *E. muelleri *beta-actin to use with a soaking method were designed with T3 or T7 RNA polymerase sequences on either end. Primer sequence for the forward primer included a T3 site (AATTAACCCTCACTAAAGG) on the 5' end followed by a beta-actin sequence of 5'-GCCCCCGAGGAGCACCCAGTCTT-3' and for the reverse primer a T7 site (TAATACGACTCACTATAGGG) with a beta-actin primer sequence of 5'-TCGGCAGTGGTGGTGAAAGAGTA-3'. PCR amplification yielded a 326-bp band, which was concentrated with a sodium acetate and ethanol precipitation. The PCR product was used as a template in RNA transcription reactions using T3 and T7 polymerase. The resulting single-stranded RNA transcripts were purified by phenol:chloroform extraction and quantified (Nanodrop). Equal amounts of T3 and T7 reactions were annealed to create double-stranded fragments by boiling for 3 minutes and then cooling slowly. dsRNA was precipitated and resuspended in 1X Strekal's before use, and dsRNA was confirmed on a 5% PAGE gel.

To produce constructs for bacterial feeding-mediated RNA interference in *E. muelleri *and *T. wilhelma *we used a protocol available from the Fire lab (http://firelab.stanford.edu/links.html) [24]. For *E. muelleri *a 290-nt product of the *Ephydatia *beta-actin gene, *Em-actb *(forward primer: 5'-GGCCCCCACTCAACCCAAAGG-3'; reverse primer: 5'CCACGCTCGGTCAGGATCTT3') was placed in the L4440 Plasmid (FireLab Vector Kit, Addgene Plasmid 1654). The L4440 (pPD129.36) vector contains two convergent T7 polymerase promoters in opposite orientation separated by a multiple cloning site [25]. In order to grow cells for expression of dsRNA and feeding experiments the expression vector was transformed into HT115(DE3) *E. coli *(which is unable to degrade dsRNA). This line was named L4440-Em-actb (abbreviated here as ds*Em-actb*). For bacterial feeding-mediated RNA interference in *T. wilhelma *a 703-bp fragment of *T. wilhelma *actin gene was placed in the L4440 plasmid. As a control, HB101 *E. coli *transformed with the pLR71 GFP constructs were fed to *E. muelleri *(results not shown). *T. wilhelma *were fed non-induced HT115(DE3)/L4440-actb *E. coli *cells as a control. Primers and constructs used for control experiments with *Em-annexin *and *Em-six *are available on request.

### RNAi-induced silencing

*E. muelleri *sponges treated with dsRNA for the soaking method were used 5-6 days after plating when the sponges have fully developed oscula (indicating that they will efficiently pump water through their tissues and feed on ingested bacteria). Strekal's culture medium was removed and replaced with dsRNA resuspended in 1X Strekal's and diluted appropriately. A range of dsRNA concentrations were used as described in the results section.

For feeding *E. muelleri *bacteria expressing dsRNA, 5 mL overnight cultures in LB broth were inoculated with L440-Em-actb one day prior to the sponge feeding stage. The next day, each overnight culture was inoculated in 100 mL LB broth with ampicillin and maintained at 37°C with shaking. Bacterial cultures were induced with 19 mg/100 mL of Isopropyl-β-D-thiogalactopyranoside (IPTG) at OD_595 _0.4 (~3 hrs after scale up) to begin bacterial production of dsRNA. Cells were induced for 1.5 hours. 50 mL of cells from each overnight culture were centrifuged in conical tubes for 8 minutes at 4000 rpm at 4°C. The supernatant was discarded and the bacterial pellet was resuspended in 50 mL 1x Strekal's media. This constituted the feeding stock. From this stock dilutions were made for each treatment to a concentration of ~1 × 10^8 ^cells/mL. The 1x Strekal's culture medium was removed from each sponge and 1 mL of the appropriate dilution of bacteria producing dsRNA was added. Sponges were cultured for 24 hours with bacteria producing dsRNA. After 24 hours, sponges were washed several times in 1X Strekal's medium, photographed on a stereomicroscope and then harvested for RNA after 1-2 hours.

For dsRNA-feeding experiments in *T. wilhelma*, a 10 ml 2xYT culture containing appropriate antibiotics was inoculated with a single colony of HT115(DE3)/L4440-actb and cultured overnight at 37°C. The culture was diluted 160-fold and allowed to grow to OD_600 _= 0.4 before IPTG was added to a final concentration of 0.4 mM in order to induce actin dsRNA expression. Incubation of the culture continued for additional 5 hours at 37°C. Cells were harvested by centrifugation and transferred to ASW immediately prior to application on *T. wilhelma*. Stage-4 buds [39] and larger specimens of *T. wilhelma *were cultured in sterile ASW containing standard 24-well cell culture plates at 24°C. On a daily basis, sponges were pulse-fed for 4.5 hours with freshly harvested induced, actin dsRNA-expressing HT115(DE3)/L4440-actb bacteria. A control group was pulse-fed with non-induced HT115(DE3)/L4440-actb bacteria. Subsequently, in order to wash out the bacteria, the total volume was exchanged twice by aerated sterile ASW on a daily basis. Sponges were sampled for actin mRNA quantification 5 hours after bacteria wash-out every 24 hours for a period of 8 days (long-term experiment) and for a period of 2 days (short-term experiment). Samples were frozen in liquid nitrogen and stored at -80°C until further batch processing. At the same time, samples were fixed for actin labeling and microscopy using 1.8% fomaldehyde and 0.2% glutaraldehyde in ASW at 4°C over night. After three washing steps (ASW), samples were dehydrated in an increasing Ethylalcohol (EtOH) series (30%, 50%, 70%) and stored in 70% EtOH until processed for microscopy.

### Time-lapse imaging

Juvenile sponges of *T. wilhelma *in the long-term silencing experiment were time-lapse imaged for 22-24 hours prior to RNA extraction. One specimen of the RNAi treatment group and one specimen of the control group were imaged simultaneously. Images were taken every minute through the bottom of the 24-well plates. Standard web cameras (Logitech, QuickCam Pro for Notebooks) attached to a Laptop were used. Cameras were controlled by the video surveillance software Active WebCam Pro (Pysoft). Analysis of body contractions was performed in ImageJ [66] using custom made macros (available upon request) as previously described [37,45].

#### QRT-PCR validation of knock down

*E. muelleri *sponges were collected after RNAi treatment and mRNA was isolated using the Qiagen RNeasy^® ^Mini Kit and treated on columns with DNase I to limit contaminating genomic DNA. Equal amounts of cDNA (125-200 ng/μl) were synthesized from experimental and control sponge mRNA using Superscript III reverse transcriptase (Invitrogen) and oligodT primer. SYBR Green (Invitrogen) chemistry and the Chromo4 (BioRad) were used for qRT-PCR using cycling conditions of 94°C for 3 min followed by 30s at 94°C, 30s at 55-61°C, 1 min at 72°C for 35 cycles with gene-specific primers (F: 5'-CGCTCTTCCCCACGCCATC-3'; R: 5'-TCGCTCGGCAGTGGTGGT-3'), which resulted in amplification of a 112 bp fragment. Control experiments using Annexin utilized primer sequences F: 5'-TCTCGTTTTGCAATCTTGGCGTAT-3'; R: 5'-GGGTGCACGTGATGAGTCTCTT-3' and for Six1/2 F: 5'-GCTTCAAGGAGAAGTCGCGTGT-3'; R: 5'-TGCCTCCGATTTTTGAACCAGT-3'. *actin, Six1/2*, and *Annexin *levels were normalized to *Ef1a*, a 'housekeeping' gene in *E. muelleri *that is consistently expressed at high levels throughout development. For all qRT-PCR experiments, duplicates were performed from master mixes. Threshold values for Ct calculation were selected by hand to optimize efficiency for all samples. Standard curves using plasmid dilutions as templates were made for each gene in each qPCR experiment and efficiency-corrected Ct values were compared to these curves to calculate relative concentrations using Opticon Monitor software. The relative concentration values of duplicates were averaged and experimental averages were normalized to Ef1a values.

*T. wilhelma *sponges were collected after RNAi treatment and homogenized with a potter. Total RNA was isolated by the RNA XS Kit (Macherey-Nagel) according to the manufacturer's instructions. cDNA was synthesized from experimental and control sponge mRNA with the First Strand cDNA Synthesis Kit (Fermentas) according to the manufacturer's instructions utilizing oligo(dt)_18 _primers. SYBR Green (KAPA SYBR Fast qPCR Mastermix) chemistry and the Smart Cycler System (Cepheid/PeqLab) were used for qRT-PCR using cycling conditions of 94°C for 3 minutes followed by 45 cycles of 94°C for 30 s, 60°C for 30 seconds, 72°C for 45 seconds with gene specific primers Act-6_U_L (5'-GCTTGAAATCCTTCCCCTTC-3') and Act-6_U_R (5'-TCTACAACCAACGCAGCAAC-3') to amplify a 92-bp fragment of the *T. wilhelma *actin gene and TW12B02-RPS5-L (5'-ACTCCACTCGTATCGGTCGT-3') and TW12B02-RPS5-R (5'-AGAGGGGACACATCAACAGC-3') to amplify a 61-bp fragment of the 40S ribosomal protein S5-related cluster (RPS5) as an internal reference for relative quantification of actin mRNA levels. For all qRT-PCR experiments, triplicates were performed from master mixes. We calculated amplification efficiencies for each replicon group and Cq values applying LinReg software (Version 12.5, http://LinRegPCR.nl) [67,68]. Efficiency-corrected relative quantification was conducted using the "efficiency-calibrated mathematical method for the relative expression ratio in real-time PCR" as used by the Roche LightCycler^® ^software [69,70].

#### Western Blot validation of knock down

Fifteen stage-five sponges were collected for each of the conditions: *dsEm-actb*, *dsEm-six*, and control-treated tissues. Lysis buffer (5% 1 M Tris pH 7.5, 6% 5 M NaCl, 0.4% 0.5 M EDTA, 0.5% Triton-X, 0.2% protease inhibitor) was added to each of the samples. The sponges were mechanically ground using an RNAse-free pestle. Protein concentrations were calculated using a BioRad assay and 15 μg of the protein were run on 12% Tris-HCl gels at 200 V at room temperature. Proteins were transferred to nitrocellulose membranes and were blocked overnight at 4°C to saturate unused protein binding sites. Blocking solution consisted of 5% nonfat dry milk in Tris Buffered Saline (TBS) buffer (pH of 7.4 and 0.01 M Tris Base, 153 mM NaCl). Membranes were washed in TBS and incubated overnight at 4°C with primary antibodies diluted to 100 μg/μl in TBST (TBS with 1% Tween 20 and 2% nonfat dry milk): Actin (h-300) rabbit polyclonal lgG or Six 1/2/4 (N-19) goat polyclonal lgG (Santa Cruz Biotechnology). The following day, nitrocellulose membranes were washed in TBST. Membranes were then incubated for 2 hours with gentle shaking at room temperature in corresponding secondary antibody: goat anti-rabbit lgG - 1:10000 FITC mouse/human absorbed or 1:10000 bovine anti-goat HRP (Santa Cruz Biotechnology). Membranes were washed in TBST and subsequently soaked in TBS for 5 minutes. Nitrocellulose membranes were detected with CDP star chemiluminescent substrate (Roche) for one minute, exposed to X-ray film and developed with an automatic film processor.

#### Phalloidin staining and evaluation of actin phenotypes

In *E. muelleri, a *protocol based on ref. [34] was utilized. *E. muelleri *gemmules were cultured and fed as above. At 22-26 hours post-feeding, sponges were fixed in 4% paraformaldehyde in PBS at 4°C overnight. Fixed sponges were washed in PBS at room temperature for 10 minutes with gentle shaking in the dark. Autofluorescence was quenched by treating with 0.1 M ammonium chloride for 5 minutes at room temperature. Tissue was permeabilized in 0.2% Triton in PBS for 10 minutes, followed by washing in cold PBS for 5 minutes. Coverslips with their attached sponges were inverted over Fastwell slides with rubber gaskets (Research Products International Corp.) containing 200 μL of BODIPY 558/568 phalloidin (Invitrogen) at a concentration of 10 U/mL. These were incubated at 3 hours in the dark at room temperature and then rinsed up to three times in PBS and mounted in fluoromount (Sigma). Fluorescence was visualized on a Leica SP2 LSCM scanning confocal microscope. Careful consideration was given to maintain settings across treatments.

In *T. wilhelma*, fixed and dehydrated samples were stepped through a decreasing series of EtOH in PBS (70%, 50%, 30%). After three washing steps in PBS, samples were incubated in 0.1% Triton X-100 in PBS for 5 minutes, followed by three washing steps in PBS. Samples were pre-incubated for 30 minutes in BSA-PBS (PBS containing 1% BSA) at 4°C followed by Alexa Fluor 488 phalloidin staining in BSA-PBS. After washing for three times in PBS, samples were mounted in ProLong Gold antifading solution. Confocal imaging was performed on a Zeiss 510 cLSM, using an Argon laser for excitation at 488 nm and a 515 nm long pass emission filter at equal settings for RNAi-treated and control specimens.

## Authors' contributions

*E. muelleri *experiments: AR adapted the feeding method, carried out molecular analyses, performed confocal microscopy analysis of phalloidin staining and co-drafted the manuscript. ED participated in the design of the study, developed the dsRNA soaking method and performed all control experiments for soaking methodology. BC made the dsRNA constructs for the feeding method, piloted feeding protocols and developed standards for qPCR. IW conducted molecular analyses and worked on optimization of feeding methods, as well as confocal microscopy analysis of phalloidin staining. DP conducted Western blot analysis for Actin and Six proteins, assayed annexin expression after RNAi, and worked extensively on manuscript revisions. SK participated in the design of both the soaking and feeding methods (including appropriate controls), provided vectors, protocols, and GFP constructs, and aided with data interpretation. MH collected *Ephydatia *tissue, conducted TEM analysis, evaluated statistical methods, and revised the manuscript. AH conceived of the study, obtained funding for the study, participated in the design and implementation of all experiments and co-drafted the manuscript. *T. wilhelma *experiments: JH adapted the feeding method, carried out molecular analyses, performed confocal microscopy analysis of phalloidin staining, prepared figure drafts and co-drafted the manuscript. KMH carried out molecular analyses and constructed the feeding vector. DVL conceived parts of the study, provided cDNA data and co-drafted the manuscript. GW provided EST data, performed the gene analysis and contributed to the manuscript. MN conceived of the study, obtained funding for the study, participated in design and implementation, performed confocal microscopy analysis, prepared final figures and co-drafted the manuscript. All authors have read and approved the final manuscript.

## Supplementary Material

Additional file 1 **Supplementary Figures.** Figure S1. *Ephydatia muelleri *and *Tethya wilhelma *actin sequences. Figure S2. *Ephydatia muelleri *and *Tethya wilhelma *dsRNA expression vector. Figure S3. Maximum likelihood (RAxML) actin gene family analysis including *Ephydatia muelleri *and *Tethya wilhelma *actin genes used for RNAi knockdown. Figure S4. Nucleotide alignment of *Tethya wilhelma *and *Ephydatia muelleri *partial actin sequences in comparison to actin of other organisms. Figure S5. Translated amino acid alignment of *Tethya wilhelma *and *Ephydatia muelleri *partial actin sequences in comparison to actin of other organisms. Figure S6. Actin dsRNA treatment by soaking method and recovery of sponge tissue after removal of actin dsRNA. Figure S7. RT-PCR and qRT-PCR analysis of dsEm-Annexin and dsEm-Six treatments. Figure S8. Western blot analysis of RNAi- and control-treated tissues. Figure S9. Viability check of RNAi-treated specimens of *T. wilhelma*. Figure S10. Cross-section of choanocyte chambers from treatment and control sponges. Figure S11. Exopinacocyte morphology of *T. wilhelma *and possible fixation artifacts demonstrated by scanning electron microscopy. Click here for file

Additional File 2 **Viability check of RNAi-treated *T. wilhelma *specimens**. Time-lapse imaging and image analysis was used to address contractility as a viability marker. Time-lapse image movie of the specimen representing the knockdown value at 201 hours in the long-term study (Figure 3C and Figure 4). The specimen is dislocated at the time of RNAi feeding due to liquid handling (time: 12:24 hh:mm; see Figure 4; culture dish had to be temporarily removed from the imaging station for feeding and was moved back afterwards). Click here for file

Additional File 3 **Nucleotide sequence alignment of contig assemblies of cytoplasmic actin complete CDS from selected Holozoa/Metazoa (fasta format)**. Click here for file

Additional File 4 **Translated amino acid sequence alignment of the complete actin CDS shown in Additional File **3**(fasta format)**. Click here for file
