# Supplementary material for: RNA interference in marine and freshwater sponges: actin knockdown in Tethya wilhelma and Ephydatia muelleri by ingested dsRNA expressing bacteria
Source: BMC Biotechnol. 2011 Jun 16;11:67. doi: 10.1186/1472-6750-11-67 (PMC3146823; doi:10.1186/1472-6750-11-67)
Supplement: Additional file 1 — Supplementary Figures. Figure S1. Ephydatia muelleri and Tethya wilhelma actin sequences. Figure S2. Ephydatia muelleri and Tethya wilhelma dsRNA expression vector. Figure S3. Maximum likelihood (RAxML) actin gene family analysis including Ephydatia muelleri and Tethya wilhelma actin genes used for RNAi knockdown. Figure S4. Nucleotide alignment of Tethya wilhelma and Ephydatia muelleri partial actin sequences in comparison to actin of other organisms. Figure S5. Translated amino acid alignment of Tethya wilhelma and Ephydatia muelleri partial actin sequences in comparison to actin of other organisms. Figure S6. Actin dsRNA treatment by soaking method and recovery of sponge tissue after removal of actin dsRNA. Figure S7. RT-PCR and qRT-PCR analysis of dsEm-Annexin and dsEm-Six treatments. Figure S8. Western blot analysis of RNAi- and control-treated tissues. Figure S9. Viability check of RNAi-treated specimens of T. wilhelma. Figure S10. Cross-section of choanocyte chambers from treatment and control sponges. Figure S11. Exopinacocyte morphology of T. wilhelma and possible fixation artifacts demonstrated by scanning electron microscopy. [file 1472-6750-11-67-S1.PDF]

Additional file 1:
Additional figures to the article

RNA interference in marine and freshwater sponges: actin knockdown in *Tethya wilhelma* and *Ephydatia muelleri* by ingested dsRNA expressing bacteria

Rivera A, Hammel JU, Haen KM, Danka E, Cieniewicz B, Winters I, Posfai D., Wörheide G, Lavrov D, Knight S, Hill M, Hill A & Nickel M

BMC Biotechnology

Additional figures:

Figure S1. *Ephydatia muelleri* and *Tethya wilhelma* actin sequences.

Figure S2. *Ephydatia muelleri* and *Tethya wilhelma* dsRNA expression vector.

Figure S3. *Maximum Likelyhood (RAxML) actin gene family analysis including Ephydatia muelleri and Tethya wilhelma actin genes used for RNAi knockdown.*

Figure S4. Nucleotide sequence alignment of *Tethya wilhelma* and *Ephydatia muelleri* actin partial sequence in comparison to actin of other organisms.

Figure S5. Translated amino acid sequence alignment of *Tethya wilhelma* and *Ephydatia muelleri* actin partial sequence in comparison to actin of other organisms.

Figure S6. Actin dsRNA treatment by soaking method and recovery of sponge tissue after removal of actin dsRNA.

Figure S7. RT-PCR and qRT-PCR analysis of dsEm-Annexin and dsEm-Six treatments.

Figure S8. Western blot analysis of RNAi and control treated tissues.

Figure S9. Viability check of RNAi treated specimens of *T. wilhelma.*

Figure S10. Cross-section of choanocyte chambers from treatment and control sponges.

Figure S11. Exopinacocyte morphology of *T. wilhelma* and possible fixation artifacts demonstrated by Scanning electron microscopy.

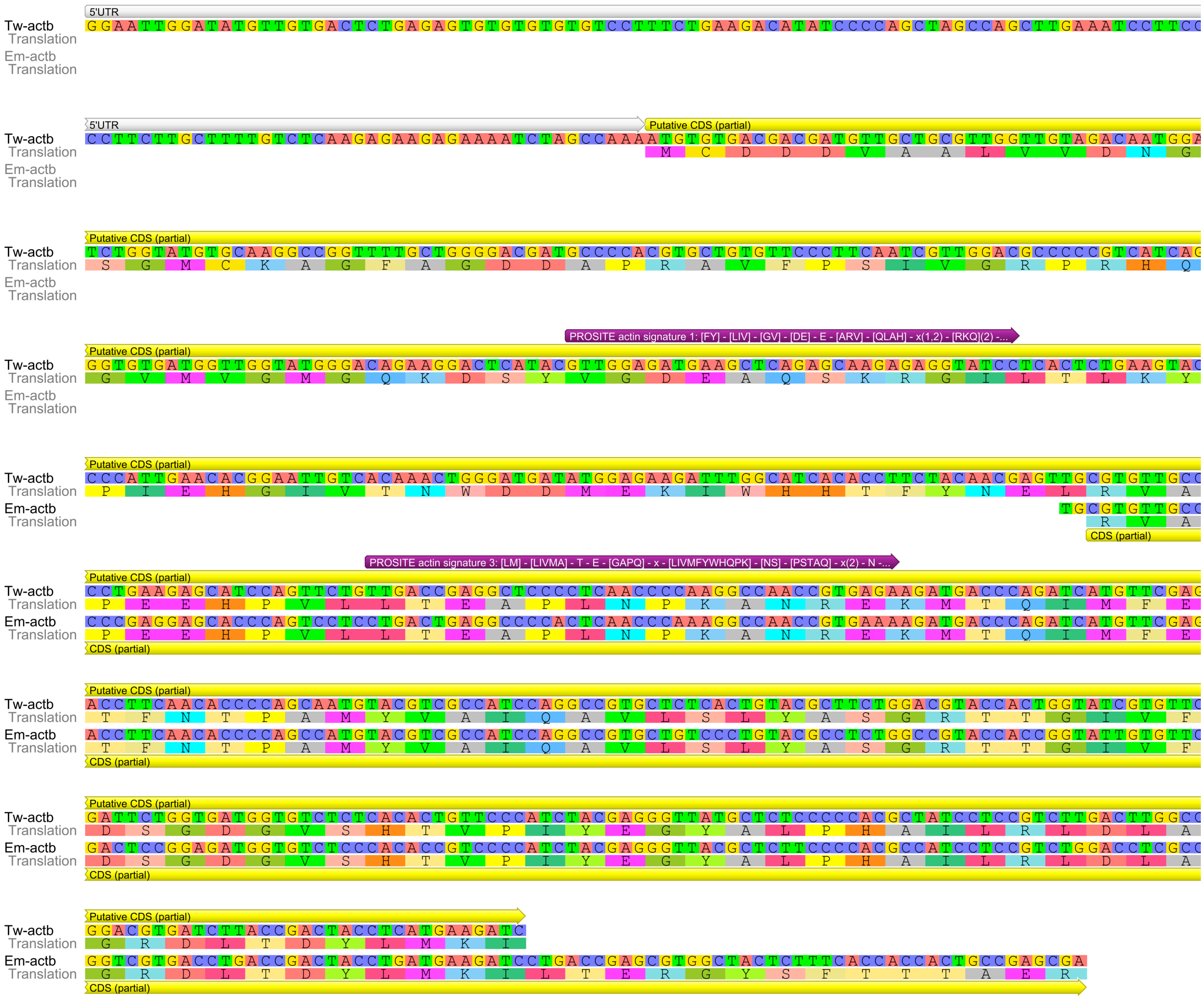

Figure S1. *Ephydatia muelleri* and *Tethya wilhelma* actin sequences. Details of the *Ephydatia muelleri* and *Tethya wilhelma* actin sequence used for RNAi experiments, aligned as nucleotide sequences and translated amino acid sequences. Coding sequences, 5'UTR and PROSITE actin motif sites are indicated.

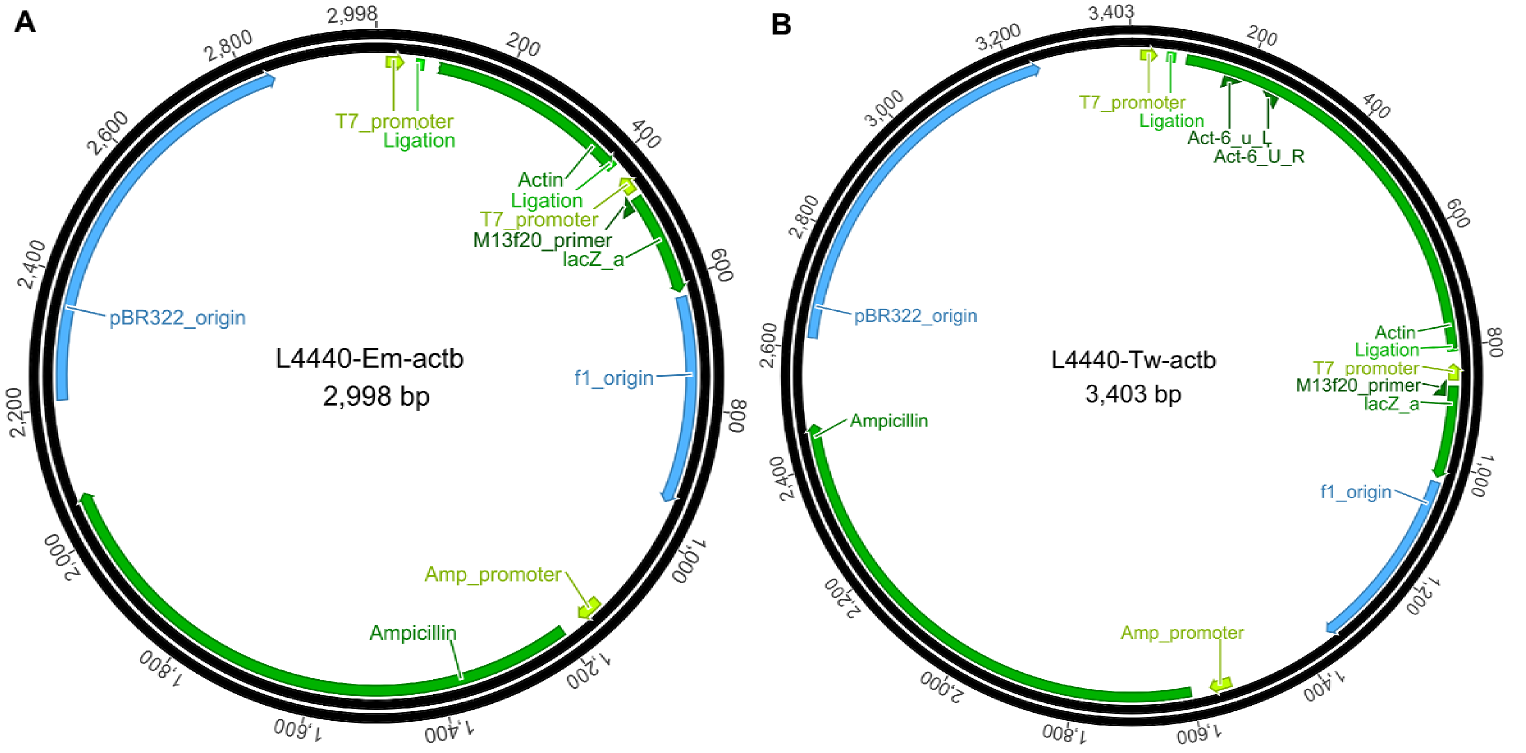

Figure S2: *Ephydatia muelleri* and *Tethya wilhelma* dsRNA expression vector. L4440-Actb vectors used to express *Ephydatia muelleri* (**A:** L4440-Em-actb, 2,998 nt) and *Tethya wilhelma* (**B:** L4440-Tw-actb, 3,403 nt) actin dsRNA in *Escherichia coli* HT115(DE3).

Figure S3: Maximum Likelyhood (RAxML) actine gene family analysis

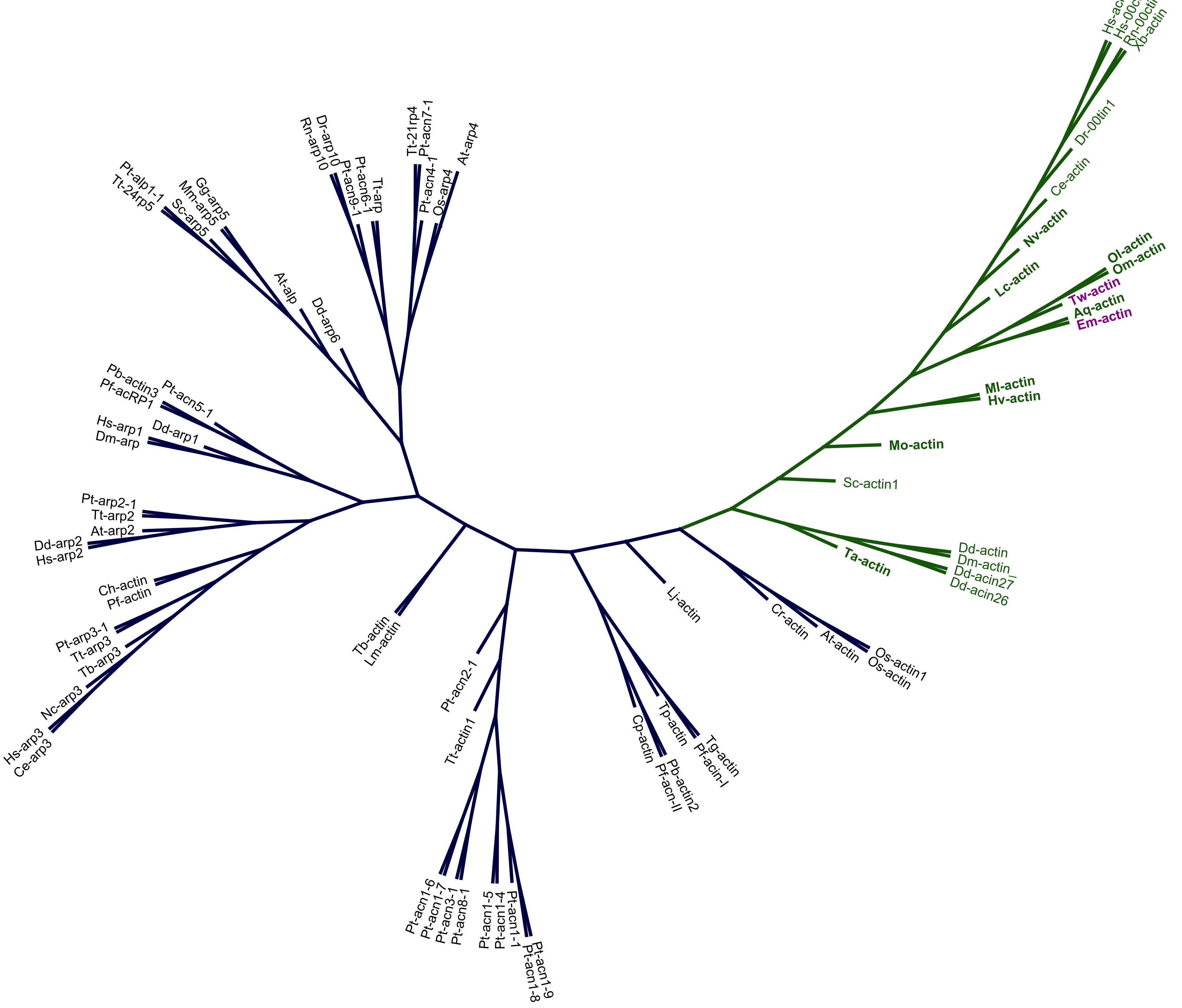

Organisms:

At: *Arabidopsis thaliana*
Aq: *Amphimedon queenslandica*
Ce: *Caenorhabditis elegans*
Ch: *Cryptosporidium hominis*
Cp: *Cryptosporidium parvum*
Cr: *Chlamydomonas reinhardtii*
Dd: *Dictyostelium discoideum*
Dm: *Drosophila melanogaster*
Dr: *Danio rerio*
Em: *Ephydatia muelleri*
Gg: *Gallus gallus*
Hs: *Homo sapiens*
Hv: *Hydra vulgaris*
Lc: *Leucetta chagoensis*
Lj: *Laminaria japonica*
Lm: *Leishmania major*
Ml: *Mnemiopsis leidyi*
Mm: *Mus musculus*
Mo: *Monosiga ovata*
Nc: *Neurospora crassa*
Nv: *Nematostella vectensis*
Oc: *Oscarella carmella*
Om: *Oopsacas minuta*
Os: *Oryza sativa*
Pb: *Plasmodium berghei*
Pf: *Plasmodium falciparum*
Pt: *Paramecium tetraurelia*
Rn: *Rattus norvegicus*
Sc: *Saccharomyces cerevisiae*
Ta: *Trichoplax adhaerens*
Tb: *Trypanosoma brucei*
Tg: *Toxoplasma gondii*
Tp: *Theileria parva*
Tt: *Tetrahymena thermophila*
Tw: *Tethya wilhelma*
Xb: *Xenopus borealis*

Figure S3. *Maximum Likelyhood (RAxML) actin gene family analysis including Ephydatia muelleri and Tethya wilhelma actin genes used for RNAi knockdown.* *Tethya wilhelma* and *Ephydatia muelleri* actins (both marked in mauve) fall into a group of Opisthokonta (i.e. Fungi + Choanoflagellata + Metazoa) cytoplasmic actins (green part of the tree), clearly separated from eukaryotic actin related proteins (ARPs) and non-opisthokont actins (blue part of the tree). Amino acid sequence alignment based on: Sehring I, Mansfeld J, Reiner C, Wagner E, Plattner H, Kissmehl R: The actin multigene family of *Paramecium tetraurelia***.** *BMC Genomics* 2007, 8:82. Eleven additional early branching metazoan EST contig based cytoplasmic actin amino acid sequences (bold), including *T. wilhelma* and *E. muelleri* have been included into the extended alignment: Aq: *Amphimedon queenslandica*; Hv: Hydra vulgaris; Em: *Ephydatia muelleri*; Lc: *Leucetta chagosensis*; Ml: *Mnemiopsis leidyi*; Mo: *Monosiga ovata*; Nv: *Nematostella vectensis*; Oc: *Oscarella carmella*; Om: *Oopsacas minuta*; Ta: *Trichoplax adhaerens*; Tw: *Tethya wilhelma*. The analysis also comprises the following organism, originally included in the alignment of Sehring et al. (2007): *Arabidopsis thaliana* (At)actin [GenBank: AAM65277], ARP2 [GenBank:AAC69601], ARP4 [GenBank: AAM53244] and ALP (listed as ARP5 in GenBank) [GenBank: BAB03145]; *Caenorhabditis elegans* (Ce) actin [GenBank: CAA34718] and ARP3 [GenBank: AAF36012]; *Chlamydomonas reinhardtii* (Cr) actin [GenBank: BAA09449]; *Cryptosporidium hominis* (Ch) actin [GenBank: XP_667340]; *Cryptosporidium parvum* (Cp) actin [GenBank: AAA28295]; *Danio rerio* (Dr)ARP1 [GenBank: NP_998537] and ARP10 [GenBank: AAH45412]; *Dictyostelium discoideum* (Dd) actin [GenBank: P02577], actin26 [GenBank:XP_646389], actin27 [GenBank: XP_636189], ARP1 [GenBank: XP_636500], ARP2 [GenBank: XP_645275] and ARP6 [GenBank:XP_637435]; *Drosophila melanogaster* (Dm) ARP [GenBank: CAA55240], actin, [GenBank: BAA20058] and actin [GenBank: AAF57294]; *Gallus gallus* (Gg) ARP5 [GenBank: NP_001008446]; *Homo sapiens* actG2[GenBank: CAG38753], ß-actin [GenBank: AAH16045], ARP1 [GenBank: AAH06372], ARP2 [GenBank:NP_005713] and ARP3 [GenBank: NP_005712]; *Laminaria japonica* (Lj) actin [GenBank: ABB80121]; *Leishmania major* (Lm) actin a [GenBank:CAC22667]; *Mus musculus* (Mm) ARP5 [GenBank: AAH52039]; *Neurospora crassa* (Nc) ARP3 [GenBank: AAC78497]; *Oryza sativa* (Os) actin1 [GenBank: XP_475316] and ARP4 [GenBank: XP_479987]; *Paramecium tetraurelia* (Pt) act1-1 [GenBank: CAD60960], act1-4 [GenBank: CAD60963], act1-5 [GenBank: CAH69678], act1-6 [GenBank: CAH03399], act1-7 [GenBank: CAH69677], act1-8 [GenBank: CAH69676], act1-9 [GenBank: CAH69752], act2-1 [GenBank: CAD60964], act3-1 [GenBank: CAD60966], act4-1 [GenBank: CAH69675], act5-1 (ARP1-1) [GenBank: CAH69674], act6-1 [GenBank: CAH69671], act7-1 (ARP4-1) GenBank: CAH74221], act8-1 [GenBank: CAH03397], act9-1 (ARP10) [GenBank: CAH69669], ALP1-1 (ARP5) [GenBank: CAH69680], ARP2-1 [GenBank: CAH69679] and ARP3-1 [GenBank: CAH74222]; *Plasmodium berghei* (Pb) actin2 [GenBank: XP_680164] and actin3 [GenBank: CAC48194]; *Plasmodium falciparum* (Pf) actin [GenBank: NP_700976], actin I [GenBank: AAA29465], actin II [GenBank: AAA29467] and actin (ARP1) [GenBank: NP_703241]; *Rattus norvegicus* (Rn) ß-actin [GenBank: ATRTC] and ARP10 [GenBank: AAH87143]; *Saccharomyces cerevisiae* (Sc) act1p [GenBank: NP_116614] and ARP5 [GenBank: CAA95933]; *Tetrahymena thermophila* (Tt) actin1 [GenBank: AAP79896], ARP [GenBank: AAN73251], ARP2 [GenBank: AAN73249] ARP3 [GenBank: AAN73250], actin family protein [GenBank: EAS01136] and actin family protein [GenBank:EAR99381]; *Theileria parva* (Tp) actin [GenBank: EAN33188]; *Toxoplasma gondii* (Tg) actin [GenBank:AAC13766]; *Trypanosoma brucei* (Tb) actin [GenBank: AAA30151] and ARP3 [GenBank: EAN76600]; and *Xenopus borealis* (Xb) actin [GenBank: CAA30390].

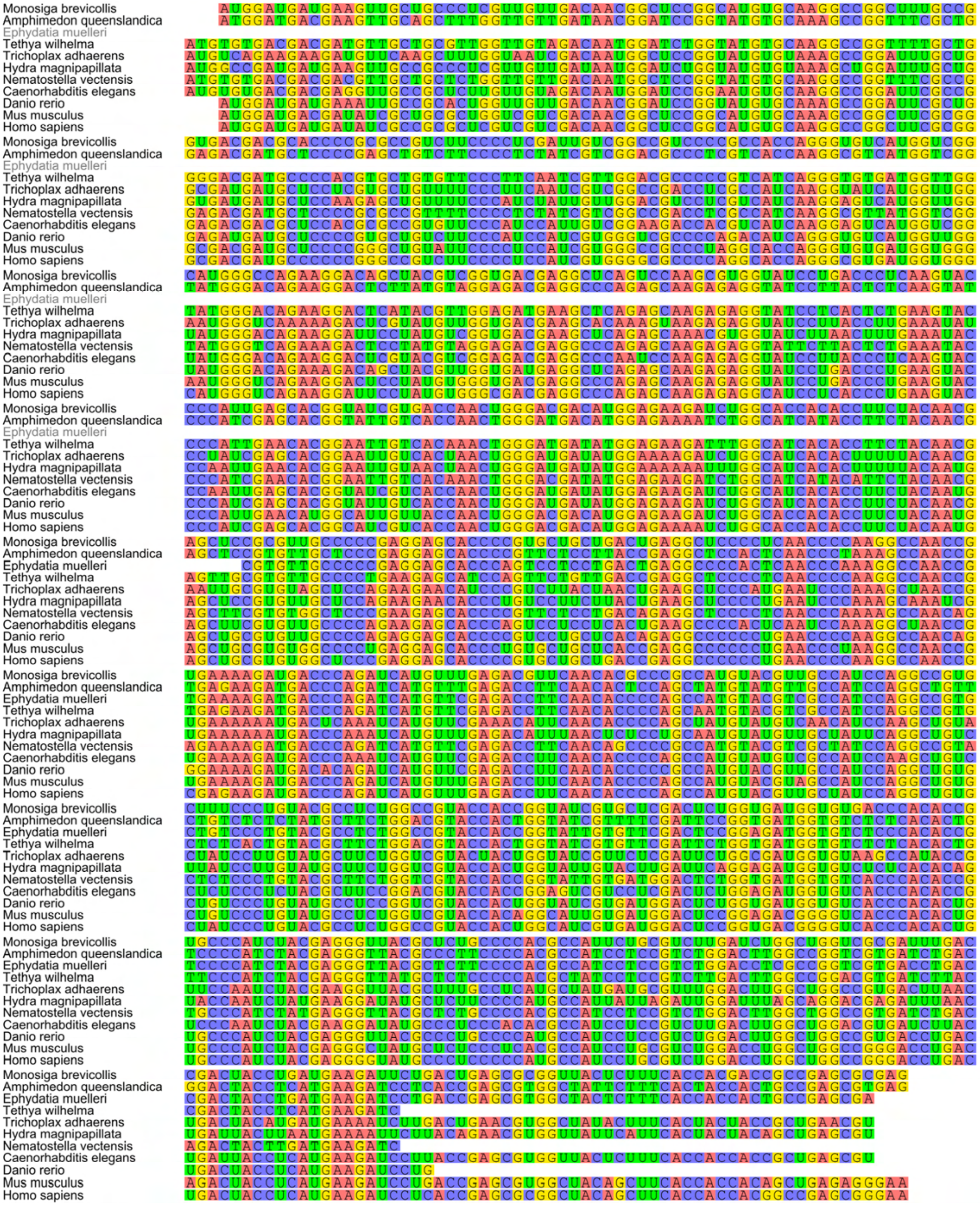

Figure S4: Nucleotide sequence alignment of *Tethya wilhelma* and *Ephydatia muelleri* actin partial sequence in comparison to actin of other organisms. Only the translated partial CDS is shown. The 5' UTR is not included in the alignment. Organisms and accession numbers: *Monosiga brevicollis* (AY026072), *Amphimedon queenslandica* (ti.1189592849), *Trichoplax adhaerens* (XM_002112678), *Hydra magnipapillata* (XM_002154426), *Nematostella vectensis* (XM_001630533), *Caenorhabditis elegans* (NM_073418), *Danio rerio* (BC154531), *Mus musculus* (NM_007393), *Homo sapiens* (NM_001101). Alignments were created in Geneious Pro 4.74 (Biomatters, Auckland, NZ) using Muscle Alignment algorithm.

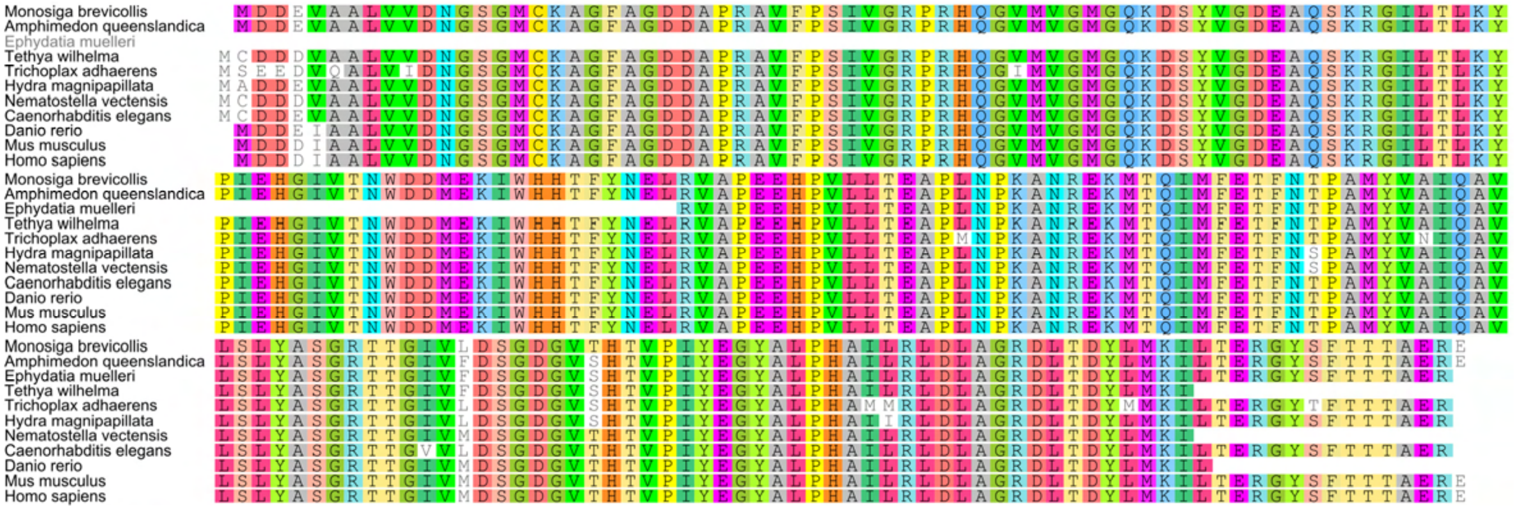

Figure S5: Translated amino acid sequence alignment of *Tethya wilhelma* and *Ephydatia muelleri* actin partial sequence in comparison to actin of other organisms. Organisms and accession numbers: *Monosiga brevicollis* (AY026072), *Amphimedon queenslandica* (ti.1189592849), *Trichoplax adhaerens* (XM_002112678), *Hydra magnipapillata* (XM_002154426), *Nematostella vectensis* (XM_001630533), *Caenorhabditis elegans* (NM_073418), *Danio rerio* (BC154531), *Mus musculus* (NM_007393), *Homo sapiens* (NM_001101). Alignments were created in Geneious Pro 4.74 (Biomatters, Auckland, NZ) using Muscle Alignment algorithm.

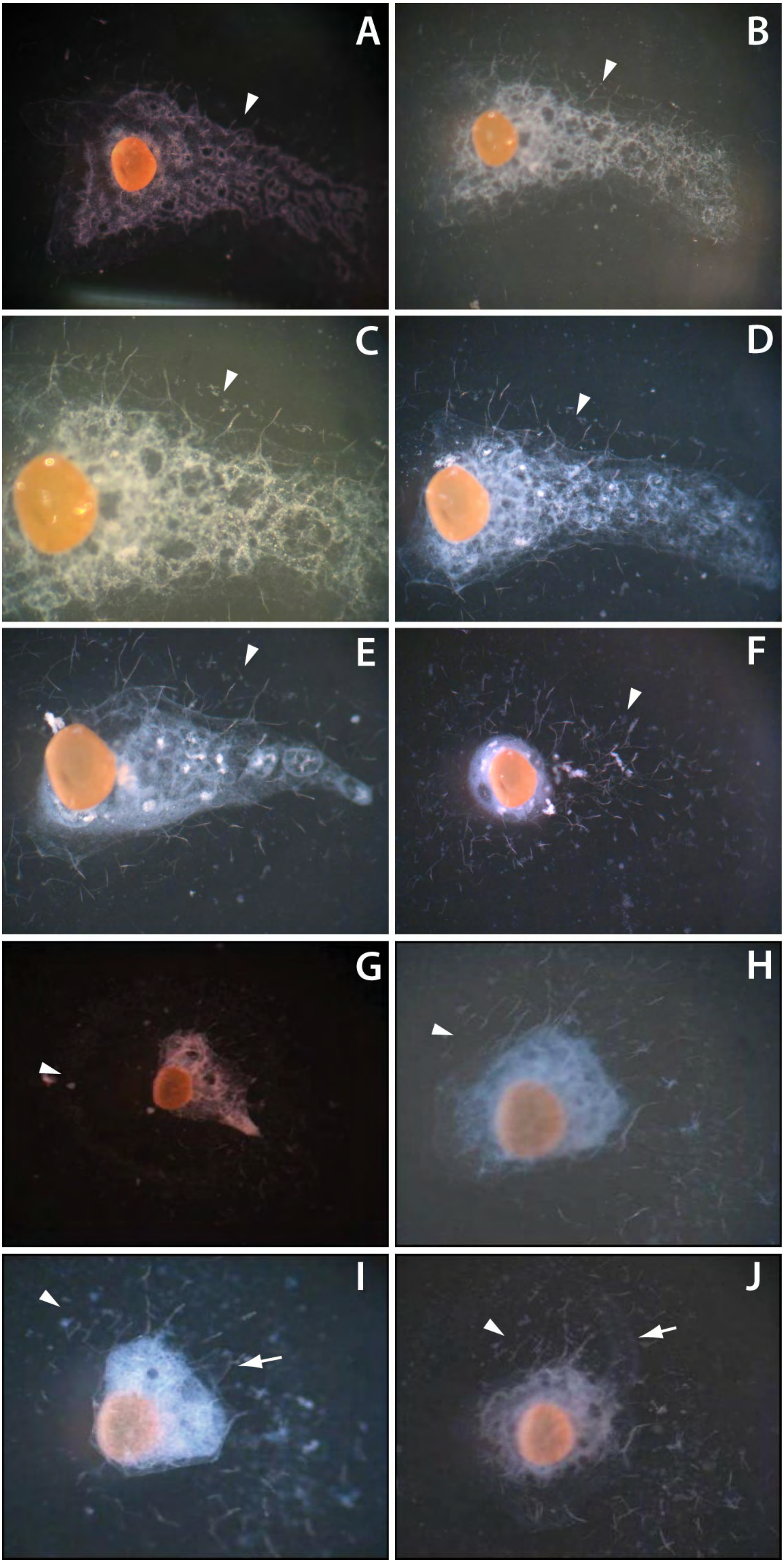

Figure S6. Actin dsRNA treatment by soaking method and recovery of sponge tissue after removal of actin dsRNA. Stage 5 *Ephydatia muelleri* treated with dsRNA for sponge beta-actin. **A.** Sponge tissue before treatment. **B.** 24 hours post treatment. **C.** Closer view of 24 hours post treatment, epithelial connections to the culture dish have receded. **D.** 48 hours post treatment, ostia less visible. **E.** 72 hours post treatment. **F.** 7 days post treatment. **G.** Seven days post treatment with dsRNA to sponge beta-actin by soaking method. **H.** 24 hours after removal of dsRNA treament. **I.** 48 hours post removal dsRNA, arrow shows developing oscula; arrowhead shows ostia. **J.** 72 hours post removal of dsRNA, arrow shows oscula and arrowheads show ostia.

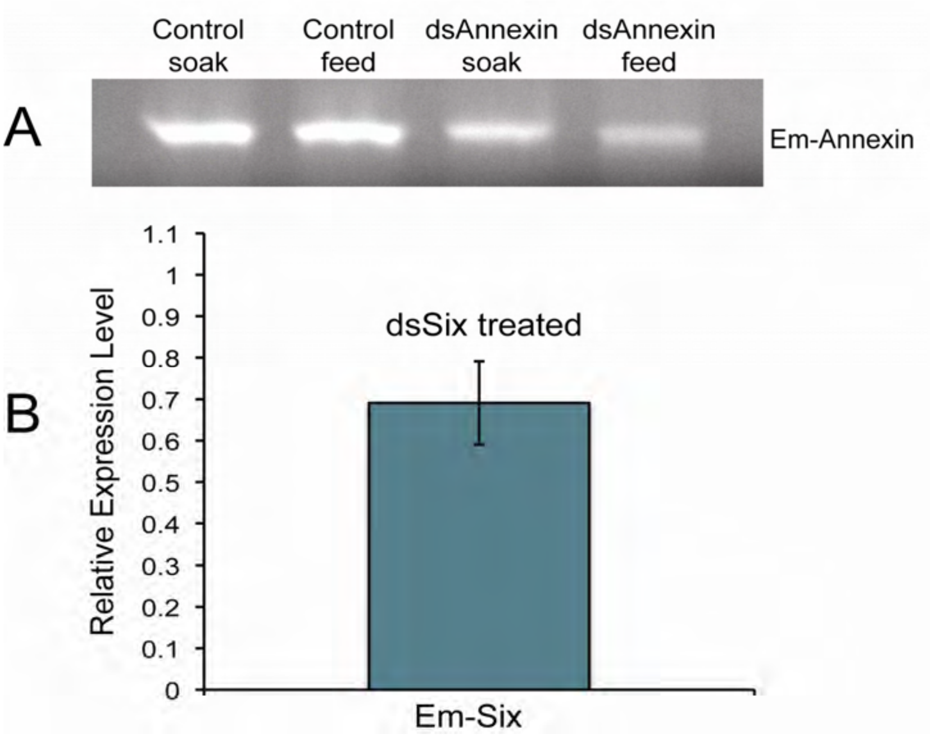

Figure S7. RT-PCR and qRT-PCR analysis of *dsEm-Annexin* and *dsEm-Six* treatments. A. Gel electrophoresis for control and *dsEm-Annexin* treated sponges using both soaking and feeding methods. Reduced expression levels are observed with both treatments. **B.** qRT-PCR analysis after dsRNA treatment with induced HT115(DE3)/L4440-*Em-six*. Levels were normalized to Ef1-alpha and gene expression levels are given as relative levels of RNAi treated sponges against control sponges.

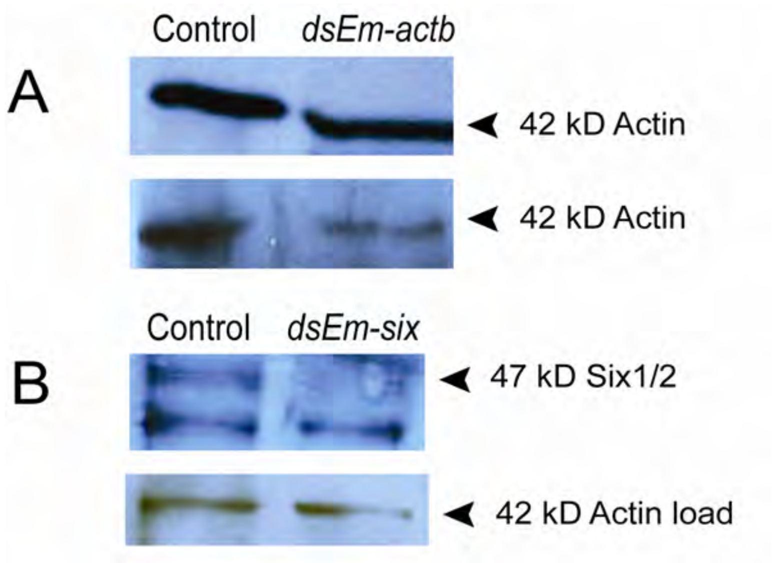

Figure S8. Western blot analysis of RNAi and control treated tissues. A. Actin protein expression for sponges fed control HT115 bacteria compared to those fed *dsEm-actb*. The upper blot shows little decrease in Actin protein levels while the lower blot shows significant reduction in Actin protein after sponges are exposed to dsRNA. A variation in knockdown from experiment to experiment was also observed by qRT-PCR. **B.** Six protein expression (upper blot) and corresponding actin loading control (lower blot) of sponges fed control bacteria compared to *dsEm-six* treated sponges.

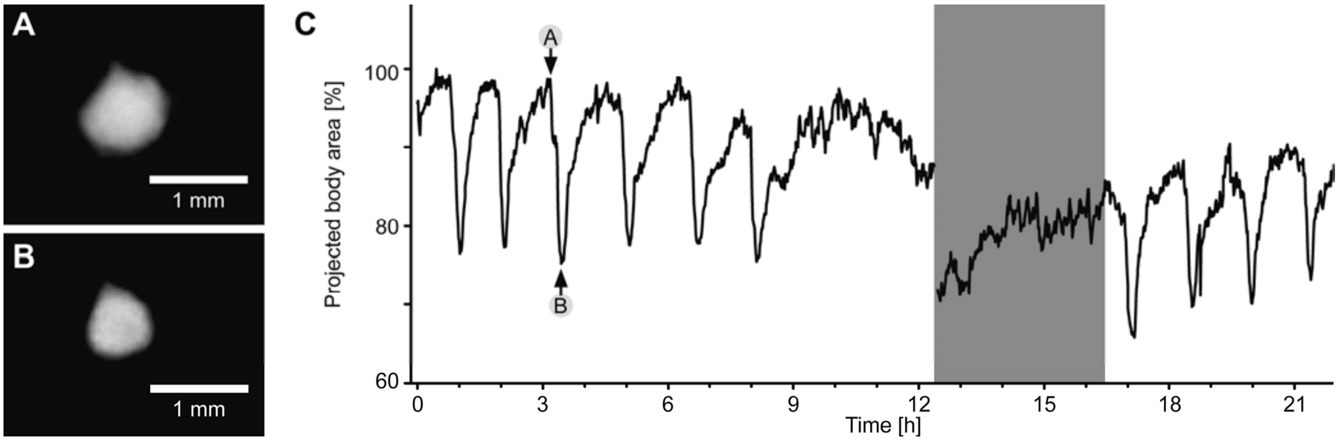

Figure S9. Viability check of RNAi treated specimens of *T. wilhelma.* Time-lapse imaging and image analysis was used to address contractility as a viability marker. Contractility is exemplified by the specimen representing the knockdown value at 201 h in the long term study (Fig. 3C), by measuring projected body areas (images taken by webcams from below through plastic culture dish, cropped to near sponge size) **A.** Expanded state (ca. 19 h prior to RNA preparation). **B.** Contracted state (ca. 18.5 h prior to RNA preparation). **C.** Contraction behavior graph. Regular contractions occur for most of the time. Grey area represents the last RNAi feeding pulse of *E.coli* HT115(DE3)/L4440-Tw-actb prior to RNA extraction. Since the suspension of bacteria alters the contrast of the images, the accuracy of the projected area measurement is restricted during the feeding pulse, but restored after bacteria wash out.

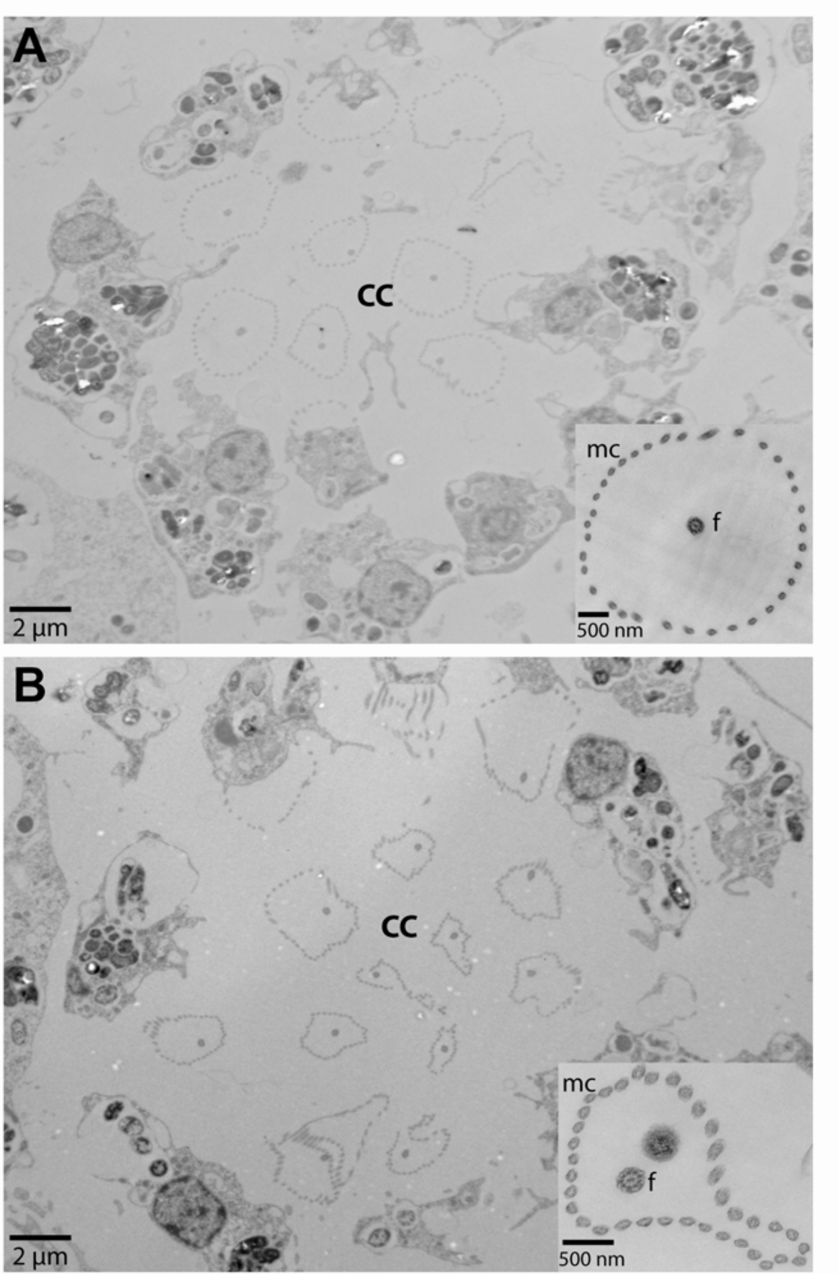

Figure S10. Cross-section of choanocyte chambers from treatment and control sponges. A. Choanocyte chamber (CC) from control treated (HT115) sponge. Inset: Microvillar collars (mc) were more consistently found to have a circular structure around the flagellum (f). B Choanocyte chamber (CC) from *dsEm-actb* treated sponge. Inset: Microvillar collars (mc) were more consistently found to have irregular (i.e., less circular) shapes around the flagellum (f).

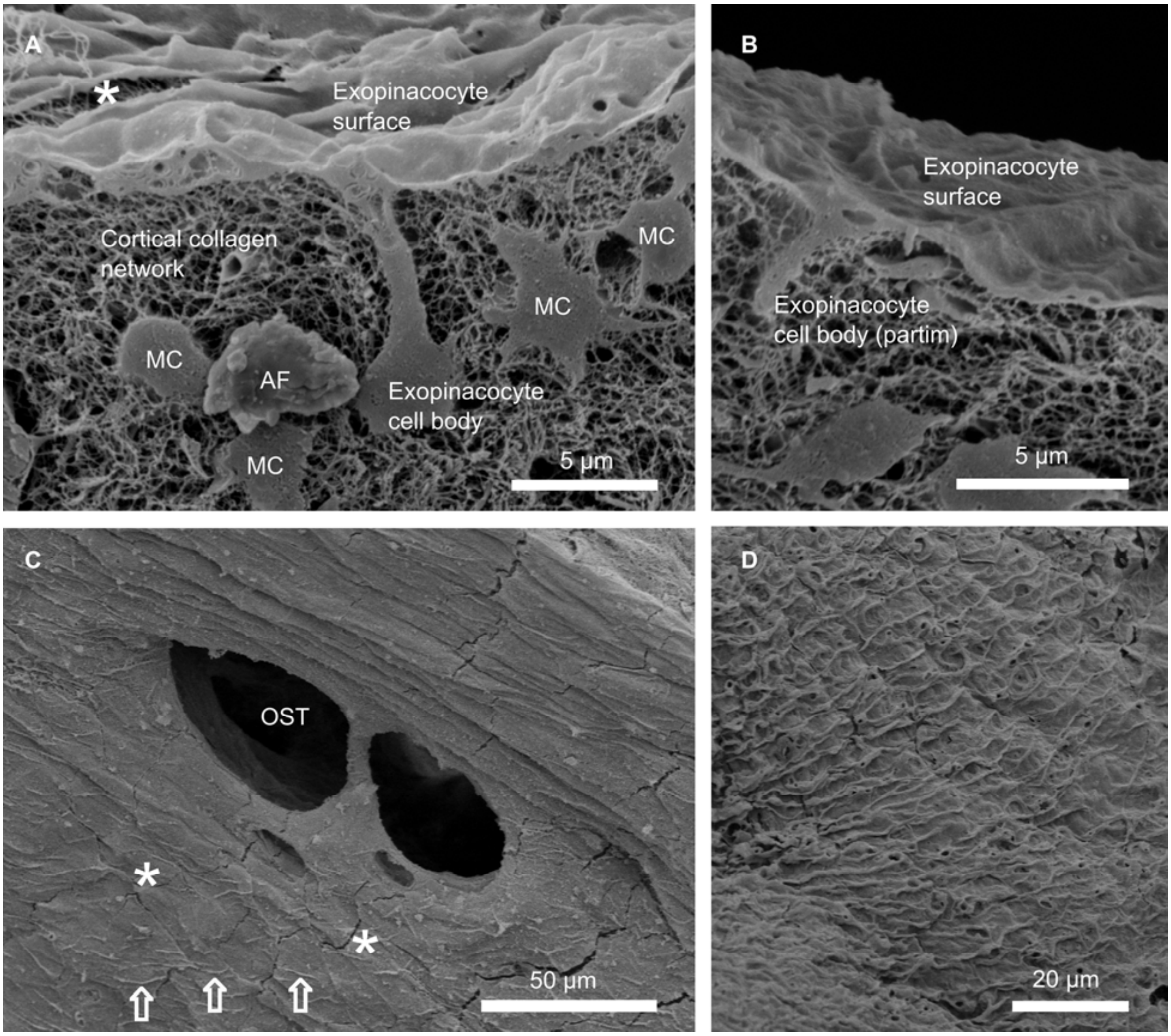

Figure S11. Exopinacocyte morphology of *T. wilhelma* and possible fixation artifacts demonstrated by Scanning electron microscopy. A./B. SEM images of sections through T-shaped or umbrella shaped exopinacocytes, sectioned where the main cell body is sunken into the cortical collagen network of the mesohyl. The outer exopinacocyte surface spans an extremely thin cover (nm-range) over the cortical mesohyl. Fixation artifacts may occur as gaps between the cells in the thin marginal pinacocyte-to-pinacocyte contact zones (asterisk). **C.** Exopinacocyte surface around two ostia openings (OST) showing typical ripple-like surface structures (arrows). Gaps between the pinacocytes (asterisks) are fixation artifacts due to moderate hypoosmotic conditions to avoid stronger shrinking artifacts. **D.** Strong shrinkage artifacts dues to moderate hyperosmotic fixation conditions. In order to avoid these artifacts we generally applied moderate hyposmotic conditions resulting in minimal artifacts as shown in A-C.
